# Virulence potential of multidrug-resistant *Acinetobacter baumannii* isolates from COVID-19 patients on mechanical ventilation: The first report from Serbia

**DOI:** 10.3389/fmicb.2023.1094184

**Published:** 2023-02-06

**Authors:** Katarina Novović, Snežana Kuzmanović Nedeljković, Mirjana Poledica, Gordana Nikolić, Bojana Grujić, Branko Jovčić, Milan Kojić, Brankica Filipić

**Affiliations:** ^1^Institute of Molecular Genetics and Genetic Engineering, University of Belgrade, Belgrade, Serbia; ^2^Institute for Medicinal Plants Research “Dr Josif Pančić”, Belgrade, Serbia; ^3^General Hospital “Dr Laza K. Lazarević” Šabac, Šabac, Serbia; ^4^Faculty of Biology, University of Belgrade, Belgrade, Serbia; ^5^Faculty of Pharmacy, University of Belgrade, Belgrade, Serbia

**Keywords:** COVID-19, ICU, *Acinetobacter baumannii*, virulence potential, AMR

## Abstract

Since the WHO declared the COVID-19 pandemic in March 2020, the disease has spread rapidly leading to overload of the health system and many of the patients infected with SARS-CoV-2 needed to be admitted to the intensive care unit (ICU). Around 10% of patients with the severe manifestation of COVID-19 need noninvasive or invasive mechanical ventilation, which represent a risk factor for *Acinetobacter baumannii* superinfection. The 64 *A. baumannii* isolates were recovered from COVID-19 patients admitted to ICU at General Hospital “Dr Laza K. Lazarević” Šabac, Serbia, during the period from December 2020 to February 2021. All patients required mechanical ventilation and mortality rate was 100%. The goal of this study was to evaluate antibiotic resistance profiles and virulence potential of *A. baumannii* isolates recovered from patients with severe form of COVID-19 who had a need for mechanical ventilation. All tested *A. baumannii* isolates (*n* = 64) were sensitive to colistin, while resistant to meropenem, imipenem, gentamicin, tobramycin, and levofloxacin according to the broth microdilution method and MDR phenotype was confirmed. In all tested isolates, representatives of international clone 2 (IC2) classified by multiplex PCR for clonal lineage identification, *bla*_AmpC_, *bla*_OXA-51_, and *bla*_OXA-23_ genes were present, as well as IS*Aba1* insertion sequence upstream of *bla*_OXA-23_. Clonal distribution of one dominant strain was found, but individual strains showed phenotypic differences in the level of antibiotic resistance, biofilm formation, and binding to mucin and motility. According to PFGE, four isolates were sequenced and antibiotic resistance genes as well as virulence factors genes were analyzed in these genomes. The results of this study represent the first report on virulence potential of MDR *A. baumannii* from hospital in Serbia.

## 1. Introduction

The outbreak of Coronavirus Disease 2019 (COVID-19) caused by severe acute respiratory syndrome coronavirus 2 (SARS-CoV-2) poses a serious threat to human health worldwide and created a perfect storm for antibiotic resistant infections in clinical settings. From the beginning of the COVID-19 pandemic, intensive care units (ICUs) have been overflowed with patients suffering severe clinical presentation of COVID-19 and acute respiratory failure, of which over 10% needed noninvasive and invasive mechanical ventilation (Zhou et al., 2020; Rouzé et al., 2021). Although mechanical ventilation is a life-saving method, it can lead to ventilator-associated pneumonia (VAP) with a high mortality rate, especially in the presence of multidrug-resistant (MDR) bacteria (e.g., *Acinetobacter baumannii*; which rapidly acquire multidrug-, extensive drug-, and even pandrug-resistance phenotypes; Harding et al., 2018; Lima et al., 2020; Sharifipour et al., 2020).

It has been confirmed that *A. baumannii* has prolonged survival and transfer in the hospital environment, such as attaching to various biotic and abiotic surfaces, including surfaces at ICUs so bacterial superinfections caused by *A. baumannii* may lead to hardly treatable conditions in COVID-19 patients (Weinberg et al., 2020).

Nowadays, the development of antibiotic resistance among *A. baumannii* strains is considered as one of the major public health concerns in hospital settings (Vázquez-López et al., 2020; Ibrahim et al., 2021).

Numerous studies indicated that *A. baumannii* isolates often exhibit a high level of resistance to almost all clinically relevant antibiotics with special emphasis on carbapenem resistance mediated by different mechanisms, such as the production of metallo-β-lactamase and oxacillinase enzymes (Runnegar et al., 2010; Rosales-Reyes et al., 2017; Amin et al., 2019).

The antibiotic resistance has increased with the use of empiric broad-spectrum antibiotic therapy to treat COVID-19 bacterial superinfections (Contou et al., 2020; Lima et al., 2020; Lukovic et al., 2020; Despotovic et al., 2021). Finally, treatment with drugs targeting IL-1 and IL-6 cytokines, as well as the use of corticosteroid therapy, might increase the risk of superinfections in COVID-19 patients (Pettit et al., 2021). *Acinetobacter baumannii* evolution during the past five decades was mainly driven by two globally disseminated clones, GC1 and GC2 (also called IC1 and IC2, IC standing for “international clone”; Holt et al., 2016).

Although many reports consider different clones or lineages of *A. baumannii* species to be associated with particular parts or regions of the world, the dramatically fast distribution of SARS-CoV-2 in 2020 has demonstrated that our knowledge regarding the spread of different pathogens is still limited (Cerezales et al., 2019; Hamidian and Nigro, 2019; Nodari et al., 2020; Shelenkov et al., 2021).

The aim of our study was to evaluate antibiotic resistance profiles and virulence potential of *A. baumannii* isolates from COVID-19 patients on mechanical ventilation (MV) admitted to ICU at the General Hospital “Dr Laza K. Lazarević” Šabac, Serbia. To the best of our knowledge, this is the first study on the virulence potential of *A. baumannii* isolates from COVID-19 patients admitted to Serbian hospitals.

## 2. Materials and methods

### 2.1. Patients

Sixty-four patients admitted to the ICU of secondary referral hospital “Dr Laza K. Lazarević” Šabac, Serbia, due to severe coronavirus disease 2019 (COVID-19) were included in this study. Patients were hospitalized during the study period from December 2020 to February 2021, and COVID-19 was confirmed by real-time PCR. All patients (*n* = 64, male:female ratio 1:1) required mechanical ventilation (MV). Besides, *Acinetobacter* spp. infection was confirmed based on bacteriological analyses of different samples recovered from COVID-19 patients on MV (Supplementary Table 1). The criteria to include patient in study protocol were (a) severe form of the COVID-19, (b) need for MV, and (c) confirmed *Acinetobacter* spp. infection. Information on patients (gender, age, and comorbidities) are presented in Supplementary Table 1. Patients admitted to ICU during the same period, without confirmed *Acinetobacter* spp. infection (*n* = 48), were not included in the study. Surveillance program through rectal swabs, was not carried out within ICU, so there is no available data if patients excluded from this study were also colonized by *A. baumannii* but did not develop the infection. The study protocol was approved by the Ethical Committee of the General hospital “Dr Laza K. Lazarević” Šabac (Approval No. 08-1/2).

### 2.2. Bacterial isolates and species identification

Bacterial isolates (one bacterial isolate per patient), belonging to *Acinetobacter* spp. (*n* = 64) were collected from blood (*n* = 28), tip of the central venous catheter (CVC; *n* = 22), tracheal aspirate (TBA; *n* = 10), tip of the aspirator (*n* = 3), and sputum (*n* = 1; Supplementary Table 1). Identification of the isolates was performed using standard microbiological procedures (cultivation, staining, and microscopy), followed by sequencing of 16S rRNA gene amplicons (Macrogen DNA sequencing service, Netherlands; Jovcic et al., 2009). 16S rRNA gene sequences were deposited to NCBI database (accession numbers ON705785–ON705830). Identification by 16S rRNA sequence was assessed using Basic Local Alignment Search Tool (BLAST, http://blast.ncbi.nlm.nih.gov/Blast.cgi) for searches against GenBank database. The strains labels were assigned randomly to preserve the anonymity of the patients (Supplementary Table 1).

### 2.3. Molecular typing

Genetic relatedness among analyzed *Acinetobacter* spp. was determined by PFGE of *Apa*I digested (3 h at 30°C) genomic DNA as previously described (Vukotic et al., 2020). Electrophoresis was run at LKB hexagonal electrode array (2015 Pulsafor unit, LKB Instruments, Bromma, Sweden) for 18 h at 300 V in 0.5 × Tris-Borate-EDTA at 9°C with pulse times of 8 s for 8 h and 18 s for 10 h during electrophoresis. Gels (1.2% agarose) were stained with ethidium bromide (SERVA Electrophoresis GmbH) and gel images were captured under UV light. Cluster analysis, using Pearson correlation coefficient with a 1.0% optimization and a hierarchic UPGMA algorithm, was used to generate a dendrogram describing the relationship among *A. baumannii* pulsotypes.

The clonal lineages determination of tested *A. baumannii* isolates was performed by multiplex PCR as previously described (Turton et al., 2007). Identification of an isolate as a member of sequence type Group 1 (international clone 2, IC2) and Group 2 (international clone 1, IC1) required amplification of all three fragments in the corresponding multiplex PCR. As positive PCR controls, *A. baumannii* strains 6077/12 and 4,031 were used for Group 1 (IC2) and Group 2 (IC1), respectively, (Novovic et al., 2015).

Multilocus sequence typing of four strains selected according to PFGE were determined using MLST 2.0 resource of Center for Genomic Epidemiology.^1^

### 2.4. Antimicrobial susceptibility testing

Antimicrobial susceptibility testing was performed using the broth microdilution method according to the European Committee on Antimicrobial Susceptibility Testing breakpoints (EUCAST, Version 11.0, 2021, https://eucast.org/). Minimum inhibitory concentrations (MICs) were determined for meropenem (1–8 μg/ml), imipenem (1–8 μg/ml), colistin (1–8 μg/ml), gentamicin (1–8 μg/ml), tobramycin (1–8 μg/ml), and levofloxacin (0.25–2 μg/ml). Experiments were done in triplicate. After 24 h incubation at 37°C, cell density was monitored by OD_570_ measurements using Multiscan FC Microplate Photometer (Thermo Scientific, United States) and MIC values were determinate as the lowest concentration of antibiotic that inhibited bacterial growth. *Acinetobacter baumannii* strain ATCC19606 was used as a control strain.

### 2.5. Molecular detection of resistance genes and virulence factor genes

The presence of the genes encoding for different oxacillinases (*bla*_OXA-23_, *bla*_OXA-24_, *bla*_OXA-51_, and *bla*_OXA-58_), cephalosporinase (*bla*_AmpC_), and metallo-β-lactamase (*bla*_NDM-1_) in genomes of analyzed isolates (*n* = 64) was monitored by PCR amplification method as previously described (Woodford et al., 2006; Beceiro et al., 2009; Teo et al., 2012). Using the same method were amplified *carO* and *omp33-36* genes (Mussi et al., 2011; Novovic et al., 2015). Localization of the insertion sequence IS*Aba1* upstream of oxacillinase genes was performed by combining a reverse primer from the pairs used for detection of each oxacillinase gene with ISAba1-F primer (Beceiro et al., 2009). Genomic DNA of four *A. baumannii* isolates was sequenced using Illumina HiSeq (MicrobesNG, IMI-School of Biosciences, University of Birmingham, Birmingham, United Kingdom). The Invitrogen Collibri Library Prep kits for Illumina HiSeq was used for the library preparation. The quality of each sequenced genome was checked using FastQC (Andrews, 2010). Genetic determinants of antibiotic resistance of four strains selected according to PFGE were determined using ResFinder 3.1. and threshold of ID = 98.00% was selected. In addition, genes determinants of virulence factors were screened in four sequenced genomes using VFDB: Virulence factors database^2^ (Chen et al., 2005). The threshold for VFDB was set at 70%. In order to analyze sugars presented in capsule, Kaptive 2.0, a tool for rapidly identifying and typing capsule (K) and outer LPS (O) loci from whole genome sequence data was used (Lam et al., 2022).

Pan-genome analysis approach was used in order to compare genomes of four sequenced *A. baumannii* isolates. Genome sequences were firstly annotated using Prokka (version 1.13) and obtained annotated GFF files were further clustered by Roary (version 3.13.0) into core genes (selection threshold for hard core genes was presence in >99% of the isolates and for soft core genes threshold was presence in 95–99% of isolates) and accessory genes (further subdivided into shell genes—present in 15–95% of isolates; and cloud genes—present in less than 15%). Phylogenetic tree was created by Roary and visualized by Phandango.

### 2.6. Biofilm formation

Collected *A. baumannii* isolates (*n* = 64) were tested for the ability to form biofilm following the protocol described previously (Stepanović et al., 2007) with slight modifications. Aliquots (20 μl) of overnight cultures adjusted to the 0.5 McFarland units, were transferred into 96-well microtiter plates containing 180 μl of Tryptone Soya Broth (TSB). Microtiter plates were incubated aerobically for 48 h at 35°C and after incubation were washed three times with phosphate-buffer saline (PBS, pH 7.2). Remaining bacteria were fixed to the surface of microtiter wells by drying plates at 65°C for 30 min. Visualization of the biofilm formation was performed by staining with 0.1% crystal violet 30 min at room temperature (RT). The excess dye was removed by washing three times with 1 × PBS. Quantification of biofilm formation was done by resolubilisation of remaining dye in mixed solution of 96% ethanol and acetone (4:1) and determination of absorbance at 570 nm using Multiscan FC Microplate Photometer (Thermo Scientific, United States). Interpretation of obtained results was performed according to recommendations of Stepanović et al. (2007) and based on previously calculated OD values for each strain. All isolates were tested in triplicate. Sterile medium tested in triplicate was used as a negative control, while *Pseudomonas aeruginosa* PAO1 strain was used as a positive control. The cut-off value (ODc) was established as three SDs above the mean OD of the negative control: ODc = average OD of negative control + (3 × SD of negative control) (Stepanović et al., 2007). ODc is calculated for each microtiter plate separately. Final OD of each tested strain was calculated as OD average of the strain minus ODc. According to calculated values, isolates were divided into four groups: no (N) biofilm producer (OD ≤ ODc), weak (W) biofilm producer (ODc < OD ≤ 2 × ODc), moderate (M) biofilm producer (2 × ODc < OD ≤ 4 × ODc), and strong (S) biofilm producer (4 × ODc < OD).

### 2.7. Mucin adhesion ability

Mucus consists primarily of water (~95%), and the major non-aqueous component is mucin, while proteoglycans, lipids, proteins, and DNA are also present in smaller quantities (Ohar et al., 2019). The binding of *A. baumannii* isolates to mucin was tested as described previously (Muñoz-Provencio et al., 2009), with the following modifications. Briefly, the wells of microtiter plates were coated with 200 μl of porcine stomach mucin type II (Sigma, Germany) resuspended in 50 mM carbonate buffer (30 mg/ml, pH 9.6). The same volume of carbonate buffer (without mucin) was added to the control wells. Plates were stored at 4°C for 48 h. Wells were washed three times with 1 × PBS, uncoated binding places were saturated with PBS containing 1% Tween 20, incubated 1 h at RT, and washed once more with 1 × PBS. Suspensions of overnight bacterial cultures were prepared in 1 × PBS and adjusted to 0.5 McFarland. Subsequently, 200 μl of each bacterial suspension was added in both coated and uncoated control wells (test was done in triplicate). After 2 h incubation at 35°C, nonadherent cells were removed using 1 × PBS with 0.05% Tween 20 and fixation of mucin-bound bacterial cells was performed by drying plate at 65°C for 1 h. 200 μl of 0.1% crystal violet (HiMedia Labs Pvt. Ltd., India) was added to the wells, plates were incubated at RT for 45 min and unbound stain was removed by three washes in 1 × PBS. Finally, citrate buffer (50 mM, pH 4) was added to dissolve the stain bound to the bacterial cells and the absorbance was measured at 570 nm using Multiscan FC Microplate Photometer (Thermo Scientific, United States).

### 2.8. Motility and gelatinase assays

All 64 *A. baumannii* isolates were tested for two types of motility: swarming and twitching. Freshly grown cultures were stabbed with sterile toothpick on the surface of modified Luria Bertani (LB) agar (tryptone—10 g/L; NaCl—5 g/L; yeast extract—5 g/L) with 0.4% agar for swarming motility and 0.8% agar for twitching motility, as described previously (Clemmer et al., 2011). *Acinetobacter baumannii* ATCC19606 strain was used as negative control as it is non-motile strain. In, addition, modified LB broth was used as a base medium for all motility assays, and plates were used on the same day that they were prepared. All isolates were tested for swarming and twitching motility under the same condition (light, temperature etc.) as it was reported that motility can be decreased in the presence of light (Mussi et al., 2010). After inoculation, the plates were incubated at 37°C for 48 h. In the case of swarming motility test, motile positive isolates were defined if the zone around the point of inoculation was greater than 10 mm, and for twitching motility isolates were characterized as non-motile (zone < 5 mm), intermediary-motile (zone 5–20 mm), or highly motile (zone ˃ 20 mm; Vijayakumar et al., 2016).

The phenotypic assay of gelatinase activity was performed as described previously (Su et al., 1991). A drop of overnight bacterial culture (10 μl) was transferred on a Gelatin agar plate containing peptone 5 g/L, yeast extract 3 g/L, gelatin 30 g/L, and agar 15 g/L (pH 7). Plates were incubated at 35°C for 48 h and after incubation flooded with a saturated solution of ammonium sulfate (550 g/L). Gelatinase activity was assessed as positive if transparent halo appeared around bacterial cells growth.

### 2.9. Statistical analyses

For comparing a difference in biofilm formation and mucin-adhesion ability among *A. baumannii* isolates obtained from different patient groups (males and females) Mann–Whitney test was used. Analyses were performed in Prism 9 version 9.3.1 and results were visualized as scatter and whicker plots. Dendrogram for analyses of the PFGE patterns was created in SPSS 28.0 for Windows.

Virulence potential data of tested isolates were summarized and visualized by a heat map created using Rx64 3.5.1 software. The results were approximated on the relative scale ranging from blue as the lowest values, progressing to white, then to red as the highest value.

## 3. Results

### 3.1. Patient’s characterization of *Acinetobacter* spp. isolates and disinfection measures in hospital

**Figure 1:**
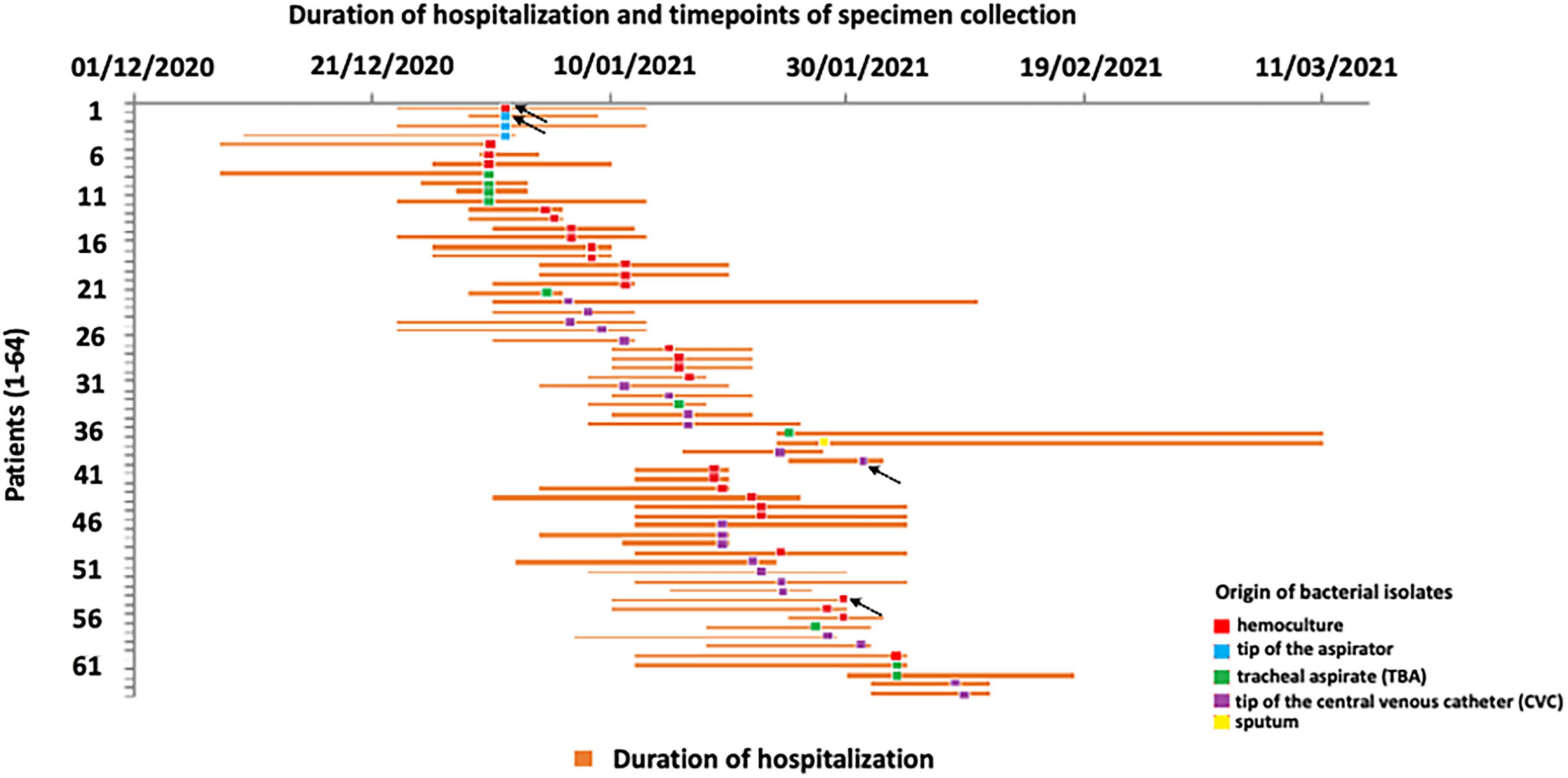
Schematic description of COVID-19 patient’s hospitalization at intensive care unit (ICU) and timepoints of *Acinetobacter baumannii* recovery. Arrows indicate the sequenced isolates.

Duration of patient hospitalization, time points of specimen collection, and origin of bacterial isolates are presented in Figure 1. All 64 isolates analyzed in this study were confirmed as *A. baumannii* according to 16S rRNA gene sequencing. According to Blast results of selected isolates the percentage of similarity of 16S rRNA genes were in the range 97.82–99.02%. The incidence of *A. baumannii* infection in COVID-19 patients was 57.14% during the study period.

Ethyl alcohol (70%) was used as disinfectant for surfaces and items which may be in contact with the patient, while chlorine-based agents were used for disinfection of floors. Healthcare workers had disposable, single use protective gloves, masks, and gowns to protect patients at ICU from possible further infection. The use of closed aspiration systems was implemented in order to prevent nosocomial infections.

The average duration of hospitalization for all patients was 16.86 days, and male COVID-19 patients were hospitalized 16.22 days on average, while female patients spent 17.5 days in the hospital. Mortality rate was 100%.

### 3.2. Genotyping analysis By PFGE and multiplex PCR

The multiplex PCR performed for identification of international clone belonging revealed that all tested *A. baumannii* isolates (*n* = 64) belong to international clone 2, IC2 (sequence type Group 1).

**Figure 2:**
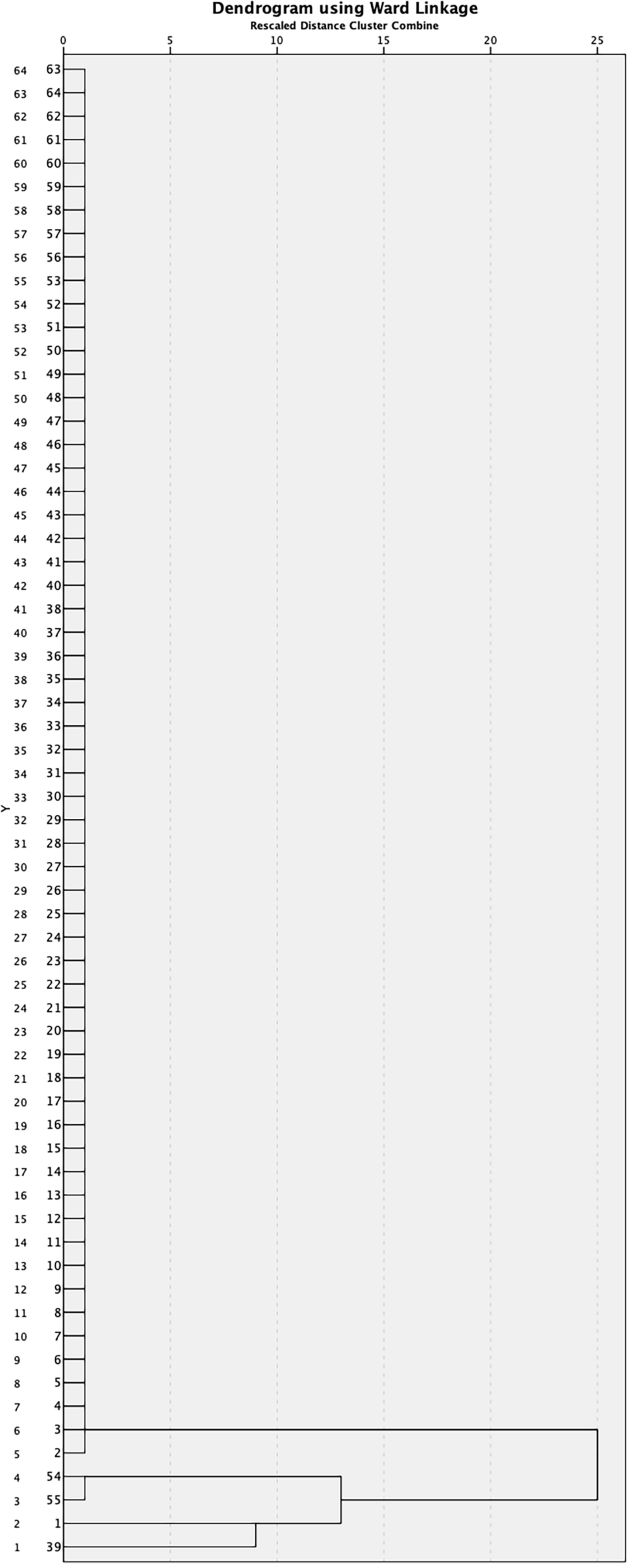
Dendrogram derived from *Apa*I PFGE patterns showing the relatedness of *Acinetobacter baumannii* isolated from COVID-19 patients in Serbia.

According to the obtained PFGE *Apa*I fingerprint, a dendrogram was constructed for all isolates and shown in Figure 2. Additionally, PFGE profiles of tested isolates obtained by *Apa*I digestion are presented in Supplementary Figure 2.

Of the 64 isolates, 60 (93.75%) possessed an identical *Apa*I profile of DNA fragments indicating the dominant presence of one pulsotype among the isolates, while only four (6.25%) isolates were different. Although these four isolates (isolates 1, 39, 54, and, 55) had a very similar *Apa*I profile to the dominant strain, they differed in at least one *Apa*I fragment; isolate 1 lacks an *Apa*I fragment of 242 kb, while a unique fragment of 228 kb is present; isolate 39 has an extra *Apa*I fragment of 250 kb that could be a plasmid, while isolates 54 and 55 have the same profile, differing from others in that they lack a fragment of 300 kb, and have a new unique fragment of 250 kb (Supplementary Figure 2). The obtained results strongly indicate the clonal distribution of the dominant strain in the hospital. Considering the slightly different genomic pattern of four isolates (differ in only one *Apa*I fragment), it can be assumed that they also originated from the dominant strain during propagation in different patients.

According to PFGE four isolates were sequenced: isolate 1, isolate 2 (as representative of all other strains), isolate 39, and, isolate 54 (as it has the same PFGE pattern as isolate 55). Sequences were deposited to NCBI databank: JAPCYJ01.1 (*A. baumannii* 1), JAPCYK01.1 (*A. baumannii* 2), JAPCYL01.1 (*A. baumannii* 39), and JAPCYM01.1 (*A. baumannii* 54).

MLST analyses revealed that all isolates were identified as ST195 (ST1816) according to Oxford nomenclature, and ST2 according to Pasteur nomenclature.

### 3.3. Resistance profiles and virulence factors of sequenced *Acinetobacter baumannii* isolates

All tested *A. baumannii* isolates (*n* = 64) were sensitive to colistin, while resistant to meropenem, imipenem, gentamicin, tobramycin, and, levofloxacin according to the broth microdilution method. According to the available data, resistance to different classes of antibiotics (carbapenem antibiotics, aminoglycosides, and, fluoroquinolone) confirmed multidrug resistance (MDR) phenotype in tested isolates (Magiorakos et al., 2012). Susceptibility of *A. baumannii* isolates was not tested to novel antibiotics, such as ceftazidime/avibactam, ceftolozane/tazobactam, and cefiderocol due to unavailability of these antibiotics. Obtained MIC values are presented in Supplementary Table 2.

Overview of antimicrobial resistance genes detected in genomes of four representative *Acinetobacter baumannii* isolates (1, 2, 39, and, 54) using ResFinder is presented in Supplementary Table 3. Screening of virulence factors genes in sequenced genomes was performed using VFDB database and results are presented in Supplementary Table 4. VFDB revealed that isolate *A. baumannii* one had slightly higher virulence potential comparing to other sequenced isolates (Supplementary Table 4). Analyses of sugar presented in capsule were performed using Kaptive 2.0 program. Obtained results revealed that there is no differences in K locus neither in O locus among sequenced genomes (Supplementary Figures 3A,B).

Pan-genome reflects the total number of genes that are present in a given dataset and the main goal of pan-genome analysis is genomic comparison of different isolates of the same species (Muzzi et al., 2007). Pan-genome analysis revealed a total of 4,096 gene clusters, which were separated into the core genome, comprised of 3,597 genes (3,597 hard core and 0 soft core genes) and accessory genome containing 499 genes in the shell and 0 genes in the cloud. The core genome of four *A. baumannii* isolates revealed that the analyzed genomes are phylogenetically related, as they share a high number of common genes (Supplementary Figure 4).

### 3.4. Molecular basis of carbapenem resistance

Typically for *A. baumannii*, intrinsic genes encoding for oxacillinase OXA-51 and cephalosporinase AmpC were detected in all tested isolates by PCR method. Additionally, all isolates gave a positive PCR signal for the *bla*_OXA-23_ gene as well as IS*Aba1* insertion sequence upstream of this gene. The gene encoding for OXA-24 oxacillinase was identified in 18 isolates (28.12%), while the *bla*_OXA-58_ gene was found in four isolates (6.25%; Supplementary Table 1). IS*Aba1* insertion sequence was not detected upstream of *bla*_OXA-24_, *bla*_OXA-51_, and, *bla*_OXA-58_ genes in analyzed isolates. The *bla*_NDM-1_ gene was not present in genomes of tested isolates. The analysis of the *carO* nucleotide sequences from tested *A. baumannii* isolates revealed that all isolates had the *carO* gene identical to corresponding gene of *A. baumannii* MS14413 (CP054302.1). Further, the gene encoding for another porin included in carbapenem resistance in *A. baumannii*, Omp33-36, of all isolates was almost identical to the *omp33-36* gene of *A. baumannii* MS14413 with only one nucleotide substitution at position 113 resulting in amino acid substitution Lys38Thr.

### 3.5. Biofilm formation

**Figure 3:**
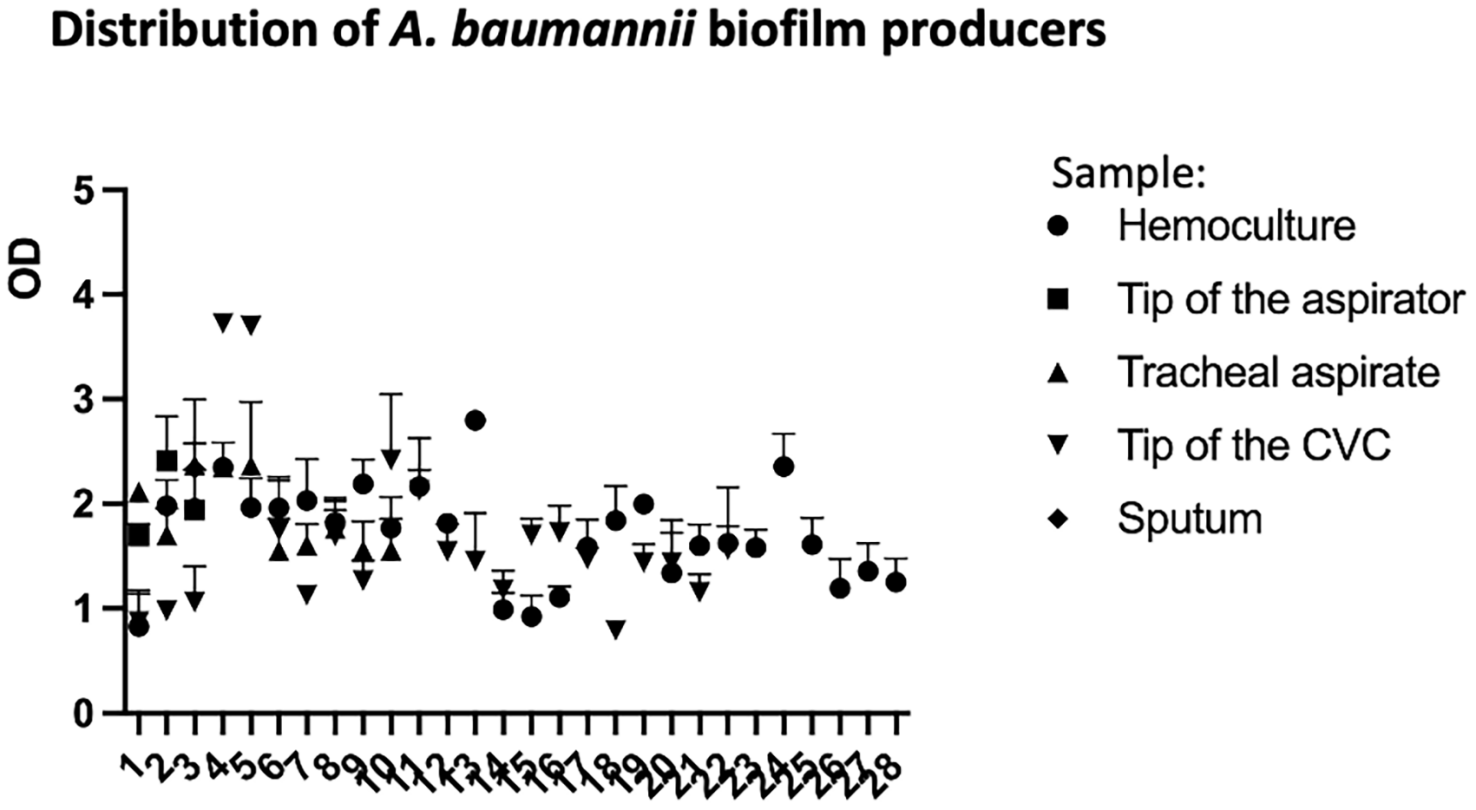
Distribution of *Acinetobacter baumannii* biofilm producers recovered from different samples (blood, tip of the aspirator, tracheal aspirate, tip of the CVC, and sputum) from COVID-19 patients.

Under the tested conditions only one isolate (1/64; 1.56%) was classified as weak (W) biofilm producer. Fifteen isolates (23.44%) were moderate (M) biofilm producers; of those 53.33% (8/15) were recovered from male and 46.67% (7/15) from female patients (Supplementary Table 5). Most of the tested isolates (75%; 48/64) were strong (S) biofilm producers recovered from female (52.08%; 25/48) and male (47.92%; 23/48). According to the Mann–Whitney test, there is no statistically significant difference in biofilm formation ability between female and male isolates. OD values of the biofilm producers by sample type (blood, tip of the aspirator, tracheal aspirate, tip of the CVC, and sputum) are presented in Figure 3.

**Figure 4:**
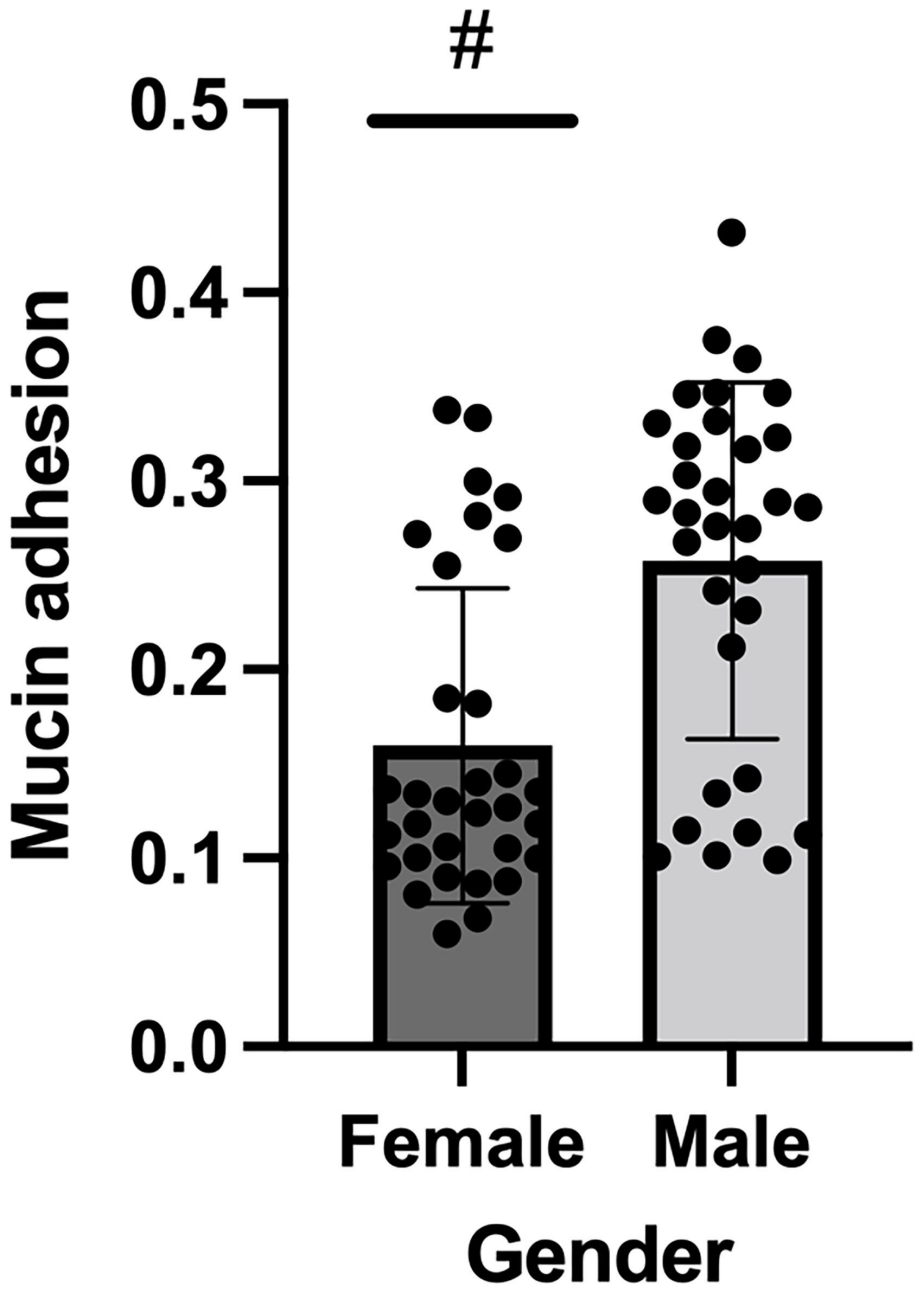
Mucin binding ability of *Acinetobacter baumannii* isolates originated from female and male COVID-19 patients; # denotes statistical significance.

Mucin-adhesion ability expressed relatively to control (non-coated wells in the microtiter plate) of 64 *A. baumannii* isolates is shown in Figure 4. The isolates recovered from female patients showed lower binding affinity to mucin compared to isolates from male patients (*p* < 0.05) according to the Mann–Whitney test.

### 3.6. Motility and gelatinase activity of tested isolates

All *A. baumannii* isolates were tested for twitching and swarming type of motility under the same temperature and light. According to twitching phenotype isolates were divided into three groups (based on the zone diameter around the inoculation point). Only 9.38% (6/64) isolates were characterized as non-motile, while the highest number of tested isolates (82.81%; 53/64) were intermediary motile. The 7.81% (5/64) isolates were highly motile, with a zone diameter up to 45 mm. Relative to the type of the sample, the number of motile strains were higher for isolates from blood (hemoculture and tip of the CVC) compared to motile isolates originated from the respiratory tract (tracheal aspirate, tip of the aspirator, and sputum, respectively) and the data are presented in Supplementary Figure 1.

Examination of swarming-like motility revealed that only 17.19% (11/64) of tested isolates were classified as a motile category. It is interesting that all isolates with the proven swarming ability, had also the ability of twitching motility, and all were originated from blood.

None of the isolates showed gelatinase activity.

### 3.7. Comparison of *Acinetobacter baumannii* isolates virulence potential

**Figure 5:**
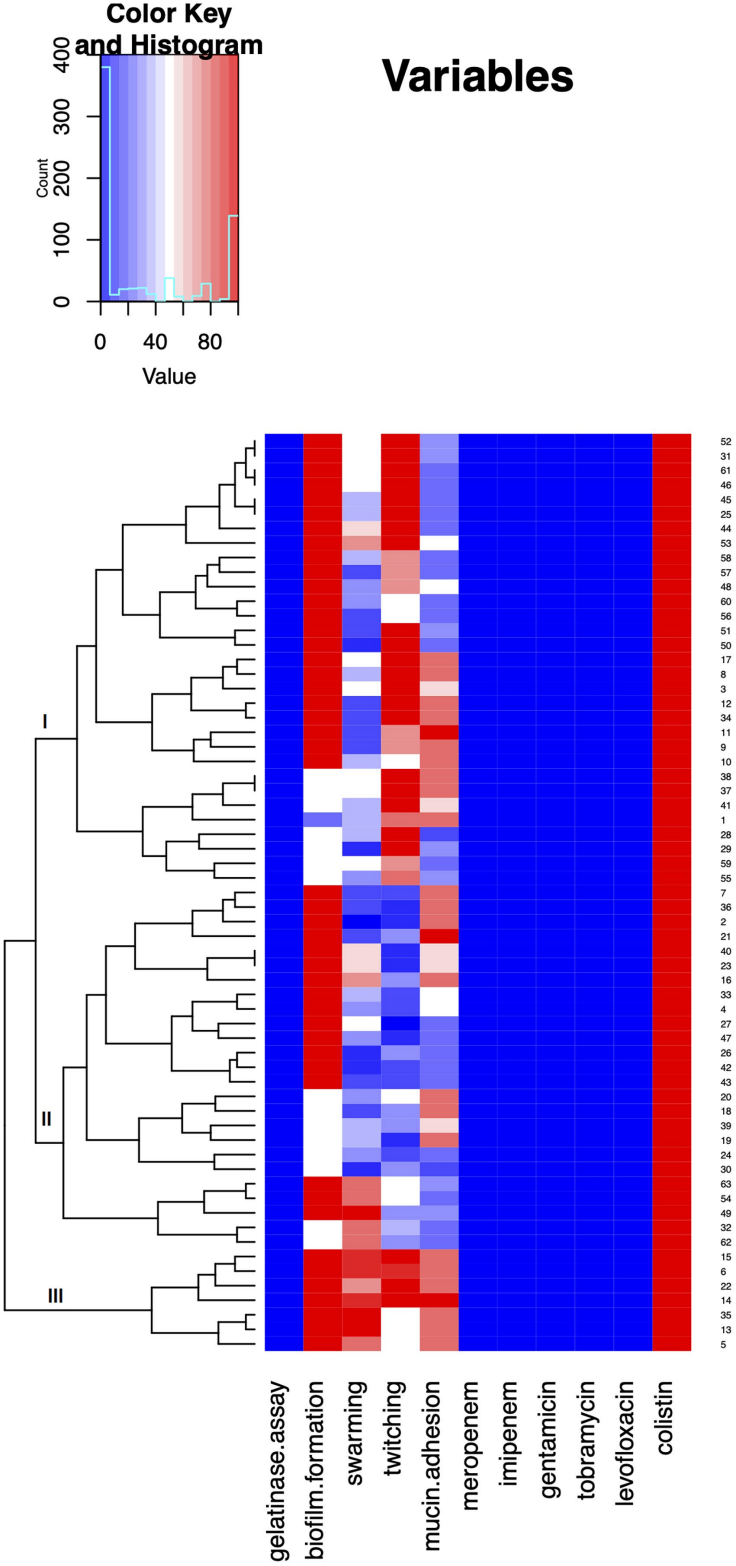
Heat map demonstrating summarized virulence potential and antibiotic resistance of tested *Acinetobacter baumannii* isolates. Results were approximated on the relative scale ranging from 0 (blue) as the lowest values, progressing to white, then to 100 (red) as the highest values.

In order to summarize the overall virulence potential, phenotypic characteristics and antibiotic resistance of 64 *A. baumannii* isolates from COVID-19 patients on MV, heat map was constructed (Figure 5). All tested isolates, according to the heat map, can be divided into three distinct clusters. Cluster three comprised the strains considered as the most virulent, including six isolates recovered from male patients (strains 5, 6, 14,15, 22, and 35) and one isolate recovered from female patient (strain 13; Figure 5). In addition, heatmap displaying the results of hierarchical clustering among 64 *A. baumannii* isolates was constructed and presented in Supplementary Figure 5.

## 4. Discussion

Epidemiology, virulence traits, and antibiotic resistance profiles of 64 *A. baumannii* isolates (32 originated from male and 32 from female patients) recovered from clinical samples of COVID-19 patients admitted to ICU who required MV were analyzed in this study.

Considering that the mortality rate of all patients was 100%, this study revealed that the duration of hospitalization of male patients (average 16.22 days) was shorter than females (average 17.5 days) what is in accordance with data that males are at higher risk of severe form of the COVID-19 and ICU admission (Peckham et al., 2020).

The genetic analysis of 64 isolates revealed an important clonal distribution of *A. baumannii* at ICU designated to COVID-19 patients. Clonal spread of *A. baumannii* highlights the importance of adopting good practices for equipment disinfection, surfaces and management of COVID-19 patients (Durán-Manuel et al., 2021).

Clonal distribution *A. baumannii* dominant hospital strain in Šabac hospital was confirmed by PFGE fingerprint analysis. Only a small number (four of 64 isolates 6.25% show low diversity in only one *Apa*I fragment) indicating that they may be derivatives of the dominant strain. According to PFGE pattern four isolates were sequenced and deposited to NCBI database. These isolates (*A. baumannii* 1, 2, 39, and 54) were analyzed for the presence of antibiotic resistance genes and genes encoding virulence factors. Genome analyses of four isolates using Virulence factors database (VFDB) revealed that isolate *A. baumannii* one has slightly higher virulence potential comparing to other sequenced isolates. MLST analyses revealed that all isolates obtained from Šabac hospital were identified as ST2 according to Pasteur nomenclature. The previous epidemiology studies in Serbia revealed that ST2 circulates in Serbian hospitals, as well as other ST types which were not detected in Šabac hospital (Novovic et al., 2015; Lukovic et al., 2020; Gajic et al., 2021; Ušjak et al., 2022).

According to Bacterial Virulence Factors Database, *A. baumannii* 1 had the highest number of genes encoding virulence factors comparing to *A. baumannii* 2, 39, and, 54. In contrast, phenotype analyses of virulence factors showed less virulence potential of *A. baumannii* 1 indicating that not all virulence genes are expressed.

Based on a more extensive study of *A. baumannii* isolates from various hospitals in Serbia (Novovic et al., 2015; Lukovic et al., 2020) have been shown to have much greater diversity of pathogenic strains compared to those detected in COVID-19 patients. These results alert us to the need for the best possible disinfection of instruments, especially mechanical ventilation devices.

Besides, the importance of adopting high standards of hygiene at ICU is the capability of *А. baumannii* to survive for long periods on biotic and abiotic surfaces by the formation of biofilms (Roca et al., 2012), and its ability to resist disinfectants and desiccation (Peleg et al., 2008). The ability to adhere and biofilm formation is assumed to be the two most important virulence factors contributing to pathogenicity of *А. baumannii* (McConnell et al., 2013).

The biofilm formation rate in *A. baumannii* is 80–91% which is higher than other species (5–24%) and represent the important virulence factor of *A. baumannii* (Amala Reena et al., 2017). Among analyzed *A. baumannii* isolates in this study even 98.44% of isolates exhibited moderate or strong ability to form biofilm with no observed statistically significant difference in biofilm formation between male and female isolates.

Data presented in this study reveal the local epidemiology of *A. baumannii* isolates characterized by a high prevalence of MDR phenotype. Several factors including high number of patients in the ICU, shortage of medical staff, challenges implementing infection prevention and control measures could be considered as risk factors for spread of MDR strains during the COVID-19 outbreak (Monnet and Harbarth, 2020; Falcone et al., 2021). High prevalence of carbapenem-resistant *A. baumannii* isolates in COVID-19 patients reported in this study is in accordance with previous studies that describe increased risk of carbapenem-resistant infection in hospitalized COVID-19 patients (Perez et al., 2020; Shinohara et al., 2021). Further, data from ICUs indicate an additional increase in prevalence of superinfections of COVID-19 patients with carbapenem-resistant *A. baumannii* (Zhang et al., 2020). As in previous studies, analyzed carbapenem-resistant *A. baumannii* isolates recovered from COVID-19 patients belonged to IC2 and harbored acquired *bla*_OXA-23_ gene alone or in combination with *bla*_OXA-24_ (Abdollahi et al., 2021; Gottesman et al., 2021). For the first time, *bla*_OXA-58_ gene, as well as IS*Aba1* upstream from *bla*_OXA-23_, were detected in *A. baumannii* isolated from COVID-19 patients. The detection of different oxacillinases in analyzed strains, as well as the presence of IS*Aba1* upstream from *bla*_OXA-23_ as a potential promoter of its expression, is of particular importance, since in that way was provided higher carbapenem resistance relative to presence of single oxacillinase (Poirel and Nordmann, 2006).

Mucus is an integral part of respiratory physiology, and it protects the respiratory tract by acting as a physical barrier against microbes. Excessive inflammation and cytokine storm can result in mucus hypersecretion in COVID-19 (Khan et al., 2021). Patients with ventilator-associated pneumonia commonly experience hyper-secretion of mucus in the respiratory tract, which normally acts as a barrier against pathogens; however, overproduction of mucus in the respiratory tract can cause patients to become more susceptible to infection with opportunistic pathogens (Dennesen et al., 2003). According to previous studies, *A. baumannii* recognizes mucin as an environmental signal, which triggers a response cascade that allows this pathogen to acquire critical nutrients and promotes host-pathogen interactions that play a role in the pathogenesis of bacterial infections (Ohneck et al., 2018). Within this study, *A. baumannii* isolates recovered from male patients showed statistically higher mucin adhesion ability compared to isolates originated from females. As mucin induces the expression of genes associated with bacterial virulence it may indicate that isolates recovered from male patients have higher virulence potential compared to isolates recovered from females in this study.

Two different types of motilities (twitching and swarming) were tested for all 64 *A. baumannii* isolates. Analyses showed that higher number of isolates exhibited twitching motility (90.62%) compared to the swarming-like motility identified in 17.10% of all isolates. We found that in the category of blood originated isolates (from hemoculture and CVC) some bacterial strains showed more proficient motility with the greater zone range, up to 45 mm, which contributes to their pathogenic profile. These highly-motile isolates were not detected in any respiratory originated samples (tracheal aspirate, tip of the aspirator, and sputum). Blood related isolates, compared to respiratory ones, stood out also by showing swarming-like motility. Motility is enabled by the presence of type IV pili, so we could hypothesize that correlation rises due to overexpression of type IV pili-related genes in isolates from blood samples compared to isolates from respiratory samples (Vijayakumar et al., 2016).

Comparison of virulence potential revealed that seven isolates (six from male and one from female) stood out from the rest in terms of analyzed phenotypes (Cluster III, Figure 5). The isolates belonging to Cluster III were strong biofilm producers and exhibited high swarming and twitching as well as mucin adhesion ability. These results might be due to the fact that men with COVID-19 are more at risk for worse outcomes and death, independent of age (Jin et al., 2020; Doerre and Doblhammer, 2022).

## 5. Conclusion

In conclusion, we reported the first study from a Serbian hospital about MDR profile and virulence potential of *A. baumannii* isolates from COVID-19 patients admitted to ICU. Our data highlight the risk to develop MDR *A. baumannii* infection during COVID-19, especially in patients on mechanical ventilation. That means that antimicrobial stewardship programs are mandatory in this population. We can emphasize that in this analysis of isolates from COVID-19 patients, clonal distribution of one strain was found, which was confirmed by PFGE analysis. Despite the presence of one dominant pulsotype, individual strains showed phenotypic differences in the level of antibiotic resistance, biofilm formation, binding to mucin and motility, which may be due to horizontal gene transfer (changes that are not detectable by PFGE), mutations or physiological adaptations. The answer to this question can be obtained by a complete analysis of the genomes, transcriptomes, and metabolomes of strains of the same pulsotype that showed different phenotypes. Considering that the mortality rate of all patients was 100%, potentially higher virulence of isolates recovered from male patients may be the reason for the shorter duration of hospitalization of male patients (average 16.22 days) comparing to females (average 17.5 days).

## Data availability statement

The datasets presented in this study can be found in online repositories. The names of the repository/repositories and accession number(s) can be found at: NCBI—JAPCYJ000000000, JAPCYK000000000, JAPCYL000000000, and JAPCYM000000000.

## Ethics statement

The studies involving human participants were reviewed and approved by General Hospital “Dr Laza K. Lazarević” Šabac, Serbia. The patients/participants provided their written informed consent to participate in this study.

## Author contributions

KN and SKN were involved in acquisition of laboratory data, analyses of data, and final approval of the manuscript. MP, GN, and BG were involved in acquisition of bacterial isolates and medical data and final approval of the manuscript. MK and BJ were involved in data analysis, critical revision of the article, and final approval of the manuscript. BF designed the study and was involved in analysis of data, drafting of article, and final approval of the manuscript. All authors contributed to the article and approved the submitted version.

## Funding

This work was funded by the Ministry of Education, Science and Technological Development of the Republic of Serbia by the Contract of implementation and financing of scientific research work Contract No: 451–03-68/2022–14/200161, 451–03-68/2022–14/200042, and 451-03-68/2022-14/200178.

## Conflict of interest

The authors declare that the research was conducted in the absence of any commercial or financial relationships that could be construed as a potential conflict of interest.

## Publisher’s note

All claims expressed in this article are solely those of the authors and do not necessarily represent those of their affiliated organizations, or those of the publisher, the editors and the reviewers. Any product that may be evaluated in this article, or claim that may be made by its manufacturer, is not guaranteed or endorsed by the publisher.

## Footnotes

^1^ www.genomicepidemiology.org

^2^ http://www.mgc.ac.cn/VFs/
