# Supplementary material for: Virulence potential of multidrug-resistant Acinetobacter baumannii isolates from COVID-19 patients on mechanical ventilation: The first report from Serbia
Source: Front Microbiol. 2023 Feb 6;14:1094184. doi: 10.3389/fmicb.2023.1094184 (PMC9941878; doi:10.3389/fmicb.2023.1094184)
Supplement: Supplementary file 1 [file Data_Sheet_1.docx]

Supplementary Material

# Supplementary Tables

**Supplementary Table 1.** Information on patients and strain labelling

| Strain number | Gender (Male/Female) | Age | Comorbidities | Sample origin | Sample collected | bla genes |  | ISAba1 bla_OXA-23_ |
| --- | --- | --- | --- | --- | --- | --- | --- | --- |
| 1 | M | >60 | BPH^1^ | hemoculture | 1-Jan-2021 | bla_OXA-23,_ bla_OXA-51_ |  | + |
| 2 | M | >60 | HTA^2^ thrombophlebitis; gonarthrosis | tip of the aspirator | 1-Jan-2021 | bla_OXA-23,_ bla_OXA-51_ |  | + |
| 3 | M | >60 | BPH | tip of the aspirator | 1-Jan-2021 | bla_OXA-23,_ bla_OXA-51_ |  | + |
| 4 | M | 40-60 | HTA; DI^3^; pulmonary fibrosis | tip of the aspirator | 1-Jan-2021 | bla_OXA-23,_  bla_OXA-24,_ bla_OXA-51_ |  | + |
| 5 | M | >60 | HTA; DI; benign brain tumour, eye tumour | hemoculture | 31-Dec-2020 | bla_OXA-23,_ bla_OXA-51_ |  | + |
| 6 | M | >60 | HTA; BPH; dementia | hemoculture | 31-Dec-2020 | bla_OXA-23,_ bla_OXA-51_ |  | + |
| 7 | M | >60 | HTA | hemoculture | 31-Dec-2020 | bla_OXA-23,_ bla_OXA-51_ |  | + |
| 8 | M | >60 | HTA; DI; benign brain tumour, eye tumour | TBA^**^ | 31-Dec-2020 | bla_OXA-23,_ bla_OXA-51_ |  | + |
| 9 | M | >60 | HTA; varicose veins; ulcus cruris; obesity | TBA^**^ | 31-Dec-2020 | bla_OXA-23,_ bla_OXA-51_ |  | + |
| 10 | M | >60 | HTA; glaucoma | TBA^**^ | 31-Dec-2020 | bla_OXA-23,_ bla_OXA-51_ |  | + |
| 11 | M | >60 | BPH | TBA^**^ | 31-Dec-2020 | bla_OXA-23,_ bla_OXA-51_ |  | + |
| 12 | F | >60 | DI | hemoculture | 4-Jan-2021 | bla_OXA-23,_ bla_OXA-51_ |  | + |
| 13 | F | >60 | DI | hemoculture | 5-Jan-2021 | bla_OXA-23,_ bla_OXA-51_ |  | + |
| 14 | M | >60 | HTA; CVA^4^; hemiplegia flaccid | hemoculture | 7-Jan-2021 | bla_OXA-23,_ bla_OXA-51_ |  | + |
| 15 | M | >60 | BPH | hemoculture | 7-Jan-2021 | bla_OXA-23,_ bla_OXA-51_ |  | + |
| 16 | M | >60 | HTA | hemoculture | 8-Jan-2021 | bla_OXA-23,_ bla_OXA-51_ |  | + |
| 17 | M | >60 | HTA | hemoculture | 8-Jan-2021 | bla_OXA-23,_ bla_OXA-51,_ bla_OXA-58_ |  | + |
| 18 | M | 40-60 | Asthma; CHF^5^; MI^6^; AoCbypass | hemoculture | 11-Jan-2021 | bla_OXA-23,_ bla_OXA-51_ |  | + |
| 19 | M | 40-60 | Asthma; CHF; MI; AoC bypass | hemoculture | 11-Jan-2021 | bla_OXA-23,_ bla_OXA-51_ |  | + |
| 20 | M | >60 | HTA; CVA; hemiplegia flaccid | hemoculture | 11-Jan-2021 | bla_OXA-23,_ bla_OXA-51_ |  | + |
| 21 | F | >60 | DI | TBA^**^ | 4-Jan-2021 | bla_OXA-23,_ bla_OXA-51_ |  | + |
| 22 | M | 40-60 | HTA; DI; myocarditis; obesity | CVC^*^ | 7-Jan-2021 | bla_OXA-23,_ bla_OXA-51_ |  | + |
| 23 | M | >60 | HTA; CVA; hemiplegia flaccid | CVC^*^ | 7-Jan-2021 | bla_OXA-23,_ bla_OXA-51_ |  | + |
| 24 | M | >60 | BPH | CVC^*^ | 7-Jan-2021 | bla_OXA-23,_ bla_OXA-51_ |  | + |
| 25 | M | >60 | BPH | CVC^*^ | 9-Jan-2021 | bla_OXA-23,_ bla_OXA-51_ |  | + |
| 26 | M | >60 | HTA; CVA; hemiplegia flaccid | CVC^*^ | 11-Jan-2021 | bla_OXA-23,_ bla_OXA-51_ |  | + |
| 27 | F | >60 | RA^7^; HTA; hysterectomy | hemoculture | 14-Jan-2021 | bla_OXA-23,_ bla_OXA-51_ |  | + |
| 28 | F | >60 | RA; HTA; hysterectomy | hemoculture | 15-Jan-2021 | bla_OXA-23,_ bla_OXA-51_ |  | + |
| 29 | F | >60 | RA; HTA; hysterectomy | hemoculture | 15-Jan-2021 | bla_OXA-23,_ bla_OXA-51_ |  | + |
| 30 | F | >60 | HTA | hemoculture | 16-Jan-2021 | bla_OXA-23,_ bla_OXA-51_ |  | + |
| 31 | M | 40-60 | Asthma; CHF; MI; AoC bypass | CVC^*^ | 11-Jan-2021 | bla_OXA-23,_ bla_OXA-51_ |  | + |
| 32 | F | >60 | RA; HTA; hysterectomy | CVC^*^ | 14-Jan-2021 | bla_OXA-23,_ bla_OXA-51_ |  | + |
| 33 | F | >60 | HTA | TBA^**^ | 15-Jan-2021 | bla_OXA-23,_ bla_OXA-51_ |  | + |
| 34 | F | >60 | RA; HTA; hysterectomy | CVC^*^ | 16-Jan-2021 | bla_OXA-23,_ bla_OXA-51_ |  | + |
| 35 | M | >60 | HTA | CVC^*^ | 16-Jan-2021 | bla_OXA-23,_ bla_OXA-51_ |  | + |
| 36 | M | >60 | AA^8^; BPH | TBA^**^ | 27-Jan-2021 | bla_OXA-23,_ bla_OXA-51_ |  | + |
| 37 | M | >60 | AA; BPH | Sputum | 29-Jan-2021 | bla_OXA-23,_  bla_OXA-24,_ bla_OXA-51_ |  | + |
| 38 | F | >60 | HTA; DI | CVC^*^ | 26-Jan-2021 | bla_OXA-23,_ bla_OXA-51_ |  | + |
| 39 | F | >60 | HTA; asthma; hypothyroidism; knee surgery; PE^9^ | CVC^*^ | 31-Jan-2021 | bla_OXA-23,_ bla_OXA-51_ |  | + |
| 40 | F | >60 | HTA; brain tumour; hysterectomy | hemoculture | 18-Jan-2021 | bla_OXA-23,_  bla_OXA-24,_ bla_OXA-51_ |  | + |
| 41 | F | >60 | HTA; brain tumour; hysterectomy | hemoculture | 18-Jan-2021 | bla_OXA-23,_ bla_OXA-51_ |  | + |
| 42 | M | 40-60 | Asthma; CHF; MI; AoC bypass | hemoculture | 19-Jan-2021 | bla_OXA-23,_ bla_OXA-51,_  bla_OXA-58_ |  | + |
| 43 | F | >60 | Varicose veins | hemoculture | 22-Jan-2021 | bla_OXA-23,_  bla_OXA-24,_ bla_OXA-51,_  bla_OXA-58_ |  | + |
| 44 | F | >60 | HTA; DI; LBBB^10^; depression | hemoculture | 23-Jan-2021 | bla_OXA-23,_ bla_OXA-51_ |  | + |
| 45 | F | >60 | HTA; DI; LBBB; depression | hemoculture | 23-Jan-2021 | bla_OXA-23,_  bla_OXA-24,_ bla_OXA-51_ |  | + |
| 46 | F | >60 | HTA; DI; LBBB; depression | CVC^*^ | 19-Jan-2021 | bla_OXA-23,_ bla_OXA-51_ |  | + |
| 47 | M | 40-60 | Asthma; CHF; MI; AoC bypass | CVC^*^ | 19-Jan-2021 | bla_OXA-23,_  bla_OXA-24,_ bla_OXA-51,_  bla_OXA-58_ |  | + |
| 48 | M | >60 | HTA; DI; BPH; HPLD^11^ | CVC^*^ | 19-Jan-2021 | bla_OXA-23,_ bla_OXA-51_ |  | + |
| 49 | F | >60 | HTA; DI; LBBB; depression | hemoculture | 25-Jan-2021 | bla_OXA-23,_  bla_OXA-24,_ bla_OXA-51_ |  | + |
| 50 | M | >60 | HTA | CVC^*^ | 22-Jan-2021 | bla_OXA-23,_  bla_OXA-24,_ bla_OXA-51_ |  | + |
| 51 | F | >60 | HTA; RA; breast cancer surgery; osteoarthrosis | CVC^*^ | 23-Jan-2021 | bla_OXA-23,_  bla_OXA-24,_ bla_OXA-51_ |  | + |
| 52 | F | >60 | HTA; DI; LBBB; depression | CVC^*^ | 25-Jan-2021 | bla_OXA-23,_  bla_OXA-24,_ bla_OXA-51_ |  | + |
| 53 | M | >60 | BPH; neo cutis | CVC^*^ | 25-Jan-2021 | bla_OXA-23,_  bla_OXA-24,_ bla_OXA-51_ |  | + |
| 54 | F | >60 | HTA; ulcus cruris | hemoculture | 30-Jan-2021 | bla_OXA-23,_ bla_OXA-24,_ bla_OXA-51_ |  | + |
| 55 | F | >60 | HTA; ulcus cruris | hemoculture | 29-Jan-2021 | bla_OXA-23,_ bla_OXA-24,_ bla_OXA-51_ |  | + |
| 56 | F | >60 | HTA; asthma; hypothyreosis; knee surgery; PE | hemoculture | 30-Jan-2021 | bla_OXA-23,_ bla_OXA-24,_ bla_OXA-51_ |  | + |
| 57 | F | >60 | HTA; COPD^12^ | TBA^**^ | 27-Jan-2021 | bla_OXA-23,_ bla_OXA-51_ |  | + |
| 58 | F | >60 | HTA; AFL^13^; osteoporosis; thyroid nodules cancer; nephrectomy; PE | CVC^*^ | 28-Jan-2021 | bla_OXA-23,_ bla_OXA-51_ |  | + |
| 59 | F | >60 | HTA; COPD | CVC^*^ | 31-Jan-2021 | bla_OXA-23,_ bla_OXA-24,_ bla_OXA-51_ |  | + |
| 60 | F | >60 | HTA; DI; LBBB; depression | hemoculture | 3-Feb-2021 | bla_OXA-23,_ bla_OXA-24,_ bla_OXA-51_ |  | + |
| 61 | F | >60 | HTA; DI; LBBB; depression | TBA^**^ | 3-Feb-2021 | bla_OXA-23,_ bla_OXA-24,_ bla_OXA-51_ |  | + |
| 62 | F | >60 | COPD; CVA; paraplegia flaccid; CMP^14^ | TBA^**^ | 3-Feb-2021 | bla_OXA-23,_ bla_OXA-51_ |  | + |
| 63 | F | >60 | HTA; DI | CVC^*^ | 8-Feb-2021 | bla_OXA-23,_ bla_OXA-24,_ bla_OXA-51_ |  | + |
| 64 | F | >60 | HTA; DI | CVC^*^ | 9-Feb-2021 | bla_OXA-23,_ bla_OXA-51_ |  | + |

BPH^1^-benign prostatic hyperplasia; HTA^2^- essential arterial hypertension; DI^3^- diabetes insipidus; CVA^4^- cerebrovascular accident; CHF^5^- congestive heart failure; MI^6^- acute myocardial infarction; RA^7^- rheumatoid arthritis; AA^8^- arrhythmia absoluta; PE^9^- pulmonary embolism; LBBB^10^- left bundle branch block; HPLD^11^- hyperlipidemia; COPD^12^- chronic obstructive pulmonary disease; AFL^13^- atrial flutter; CMP^14^- cardiomyopathy; TBA^*^- tracheal aspirate; CVC^**^- tip of the central venous catheter

**Supplementary Table 2.** Minimal inhibitory concentrations (MICs) for different antibiotics. EUCAST breakpoints were used for MIC interpretation.

| Strain number | **Colistin^a^** | | **Gentamicin^b^** | | **Imipenem^c^** | | **Meropenem^d^** | | **Levofloxacin^e^** | | **Tobramycin^f^** | |
| --- | --- | --- | --- | --- | --- | --- | --- | --- | --- | --- | --- | --- |
|  | MIC (μg/ml) | Category | MIC (μg/ml) | Category | MIC (μg/ml) | Category | MIC (μg/ml) | Category | MIC (μg/ml) | Category | MIC (μg/ml) | Category |
| 1 | 2 | S | >8 | R | >8 | R | >8 | R | >2 | R | 8 | R |
| 2 | 2 | S | >8 | R | 8 | R | >8 | R | >2 | R | >8 | R |
| 3 | 2 | S | >8 | R | 8 | R | >8 | R | >2 | R | >8 | R |
| 4 | 2 | S | >8 | R | 8 | R | >8 | R | >2 | R | >8 | R |
| 5 | 2 | S | >8 | R | 8 | R | >8 | R | >2 | R | >8 | R |
| 6 | 2 | S | >8 | R | 8 | R | >8 | R | >2 | R | >8 | R |
| 7 | 2 | S | >8 | R | >8 | R | >8 | R | >2 | R | >8 | R |
| 8 | 2 | S | >8 | R | >8 | R | >8 | R | >2 | R | >8 | R |
| 9 | 2 | S | >8 | R | >8 | R | >8 | R | >2 | R | >8 | R |
| 10 | 2 | S | >8 | R | >8 | R | >8 | R | >2 | R | >8 | R |
| 11 | 2 | S | >8 | R | >8 | R | >8 | R | >2 | R | >8 | R |
| 12 | 2 | S | >8 | R | >8 | R | >8 | R | >2 | R | >8 | R |
| 13 | 2 | S | >8 | R | 8 | R | >8 | R | >2 | R | >8 | R |
| 14 | 2 | S | >8 | R | >8 | R | >8 | R | >2 | R | >8 | R |
| 15 | 2 | S | >8 | R | >8 | R | >8 | R | >2 | R | >8 | R |
| 16 | 2 | S | >8 | R | >8 | R | >8 | R | >2 | R | >8 | R |
| 17 | 2 | S | >8 | R | 8 | R | >8 | R | >2 | R | >8 | R |
| 18 | 2 | S | >8 | R | 8 | R | >8 | R | >2 | R | >8 | R |
| 19 | 2 | S | >8 | R | 8 | R | >8 | R | >2 | R | >8 | R |
| 20 | 2 | S | >8 | R | 8 | R | >8 | R | >2 | R | >8 | R |
| 21 | 2 | S | >8 | R | 8 | R | >8 | R | >2 | R | >8 | R |
| 22 | 2 | S | >8 | R | 8 | R | >8 | R | >2 | R | >8 | R |
| 23 | 2 | S | >8 | R | 8 | R | >8 | R | >2 | R | >8 | R |
| 24 | 1 | S | >8 | R | 8 | R | >8 | R | >2 | R | >8 | R |
| 25 | 2 | S | >8 | R | 8 | R | >8 | R | >2 | R | >8 | R |
| 26 | 2 | S | >8 | R | 8 | R | >8 | R | >2 | R | >8 | R |
| 27 | 2 | S | >8 | R | 8 | R | >8 | R | >2 | R | >8 | R |
| 28 | 2 | S | >8 | R | 8 | R | >8 | R | >2 | R | >8 | R |
| 29 | 2 | S | >8 | R | 8 | R | >8 | R | >2 | R | >8 | R |
| 30 | 2 | S | >8 | R | 8 | R | >8 | R | >2 | R | >8 | R |
| 31 | 2 | S | >8 | R | 8 | R | >8 | R | >2 | R | >8 | R |
| 32 | 2 | S | >8 | R | 8 | R | >8 | R | >2 | R | >8 | R |
| 33 | 2 | S | >8 | R | >8 | R | >8 | R | >2 | R | >8 | R |
| 34 | 2 | S | >8 | R | >8 | R | >8 | R | >2 | R | >8 | R |
| 35 | 2 | S | >8 | R | 8 | R | >8 | R | >2 | R | >8 | R |
| 36 | 2 | S | >8 | R | >8 | R | >8 | R | >2 | R | >8 | R |
| 37 | 2 | S | >8 | R | 8 | R | >8 | R | >2 | R | >8 | R |
| 38 | 2 | S | >8 | R | >8 | R | >8 | R | >2 | R | >8 | R |
| 39 | 2 | S | >8 | R | >8 | R | >8 | R | >2 | R | >8 | R |
| 40 | 2 | S | >8 | R | >8 | R | >8 | R | >2 | R | >8 | R |
| 41 | 2 | S | >8 | R | >8 | R | >8 | R | >2 | R | >8 | R |
| 42 | 2 | S | >8 | R | >8 | R | >8 | R | >2 | R | >8 | R |
| 43 | 2 | S | >8 | R | 8 | R | >8 | R | >2 | R | >8 | R |
| 44 | 2 | S | >8 | R | >8 | R | >8 | R | >2 | R | >8 | R |
| 45 | 2 | S | >8 | R | 8 | R | >8 | R | >2 | R | >8 | R |
| 46 | 2 | S | >8 | R | 8 | R | >8 | R | >2 | R | >8 | R |
| 47 | 2 | S | >8 | R | 8 | R | >8 | R | >2 | R | >8 | R |
| 48 | 2 | S | >8 | R | >8 | R | >8 | R | >2 | R | >8 | R |
| 49 | 2 | S | >8 | R | >8 | R | >8 | R | >2 | R | >8 | R |
| 50 | 2 | S | >8 | R | >8 | R | >8 | R | >2 | R | >8 | R |
| 51 | 2 | S | >8 | R | >8 | R | >8 | R | >2 | R | >8 | R |
| 52 | 2 | S | >8 | R | >8 | R | >8 | R | >2 | R | >8 | R |
| 53 | 2 | S | >8 | R | >8 | R | >8 | R | >2 | R | >8 | R |
| 54 | 2 | S | >8 | R | >8 | R | >8 | R | >2 | R | >8 | R |
| 55 | 2 | S | >8 | R | >8 | R | >8 | R | >2 | R | >8 | R |
| 56 | 2 | S | >8 | R | >8 | R | >8 | R | >2 | R | >8 | R |
| 57 | 2 | S | >8 | R | >8 | R | >8 | R | >2 | R | >8 | R |
| 58 | 2 | S | >8 | R | >8 | R | >8 | R | >2 | R | >8 | R |
| 59 | 2 | S | >8 | R | >8 | R | >8 | R | >2 | R | >8 | R |
| 60 | 2 | S | >8 | R | 8 | R | >8 | R | >2 | R | >8 | R |
| 61 | 2 | S | >8 | R | 8 | R | >8 | R | >2 | R | >8 | R |
| 62 | 2 | S | >8 | R | >8 | R | >8 | R | >2 | R | >8 | R |
| 63 | 2 | S | >8 | R | 8 | R | >8 | R | >2 | R | >8 | R |
| 64 | 2 | S | >8 | R | >8 | R | >8 | R | >2 | R | >8 | R |
| a: Breakpoints for Colistin (S≤2, R˃2); b: Breakpoint sfor Gentamicin (S≤4, R˃4); c: Breakpoints for Imipenem (S≤2, R˃4); d: Breakpoints for Meropenem (S≤2, R˃2), e: Breakpoints for Levofloxacin (S≤0.5, R˃1); f: Breakpoints for Tobramycin (S≤4, R˃4) | | | | | | | | | | | | |

**Supplementary Table 3.** Overview of antimicrobial resistance genes detected in genomes of four representative A*. baumannii* isolates using ResFinder.

| Strain | Antimicrobial resistance genes |
| --- | --- |
| JAPCYJ01.1 (*A. baumannii* 1) | *sul1*, *qacE* , *aac(3)-Ia*, *aadA1*, *catA1*, *bla*_OXA-66_, *bla*_ADC-25_, *bla*_OXA-23_ |
| JAPCYK01.1 (*A. baumannii* 2) | *sul1*, *mph*(E), *msr*(E), *qacE* , *aac(3)-Ia*, *aadA1*, *armA*, *catA1*, *bla*_OXA-66_, *bla*_ADC-25_, *bla*_OXA-23_ |
| JAPCYL01.1 (*A. baumannii* 39) | *sul1*, *mph*(E), *msr*(E), *qacE* , *aac(3)-Ia*, *aph(3')-VIa*, *aadA1*, *armA*, *catA1*, *bla*_OXA-66_, *bla*_ADC-25_, *bla*_OXA-23_ |
| JAPCYM01.1 (*A. baumannii* 54) | *sul1*, *mph*(E), *msr*(E), *qacE* , *aac(3)-Ia*, *aadA1*, *armA*, *catA1*, *bla*_OXA-66_, *bla*_ADC-25_, *bla*_OXA-23_ |

**Supplementary Table 4.** Virulence factors genes detected in sequenced genoms using VFDB: Virulence Factors Database

| VFclass | Virulence factors | Related genes | *A. baumannii* 1 | *A. baumannii* 2 | *A. baumannii* 39 | *A. baumannii* 54 |
| --- | --- | --- | --- | --- | --- | --- |
| Adherence | Outer membrane protein | *ompA* | + | + | + | + |
|  | Fibronectin binding protein | *fbpA* | + | - | - | - |
|  | Flagella | *fliP* | + | - | - | - |
|  | GroEL | *groEL* | + | - | - | - |
|  | Polar flagella | *flmH* | + | - | - | - |
|  | Streptococcal plasmin receptor/GAPDH | *Plr/gapA* | + | - | - | - |
| Biofilm formation | AdeFGH efflux pump/transport autoinducer | *adeF* | + | + | + | + |
|  |  | *adeG* | + | + | + | + |
|  |  | *adeH* | + | + | + | + |
|  | Biofilm-associated protein | *bap* | + | + | + | + |
|  | Csu pili | *csuA/B* | - | + | + | + |
|  |  | *csuA* | - | + | + | + |
|  |  | *csuB* | - | + | + | + |
|  |  | *csuC* | - | + | + | + |
|  |  | *csuD* | - | + | + | + |
|  |  | *csuE* | - | + | + | + |
|  | PNAG (Polysaccharide poly-N-acetylglucoseamine | *pgaA* | + | + | + | + |
|  |  | *pgaB* | + | + | + | + |
|  |  | *pgaC* | + | + | + | + |
|  |  | *pgaD* | + | + | + | + |
| Enzyme | Phospholopase C | *plc* | + | + | + | + |
|  | Phospholipase D | *plcD* | + | + | + | + |
| Immune evasion | LPS | *lpsB* | + | + | + | + |
|  |  | *lpxA* | + | + | + | + |
|  |  | *lpxC* | + | + | + | + |
|  |  | *lpxD* | + | + | + | + |
|  |  | *lpxL* | + | + | + | + |
|  |  | *IpxM* | + | + | + | + |
| Iron uptake | Acinetobactin | *barA* | + | + | + | + |
|  |  | *barB* | + | + | + | + |
|  |  | *basA* | + | + | + | + |
|  |  | *basB* | + | + | + | + |
|  |  | *basC* | + | + | + | + |
|  |  | *basD* | + | + | + | + |
|  |  | *basF* | + | + | + | + |
|  |  | *basG* | + | + | + | + |
|  |  | *basH* | + | + | + | + |
|  |  | *basI* | + | + | + | + |
|  |  | *basJ* | + | + | + | + |
|  |  | *bauA* | + | + | + | + |
|  |  | *bauB* | + | + | + | + |
|  |  | *bauC* | + | + | + | + |
|  |  | *bauD* | + | + | + | + |
|  |  | *bauE* | + | + | + | + |
|  |  | *bauF* | + | + | + | + |
|  |  | *entE* | + | + | + | + |
|  | Heme utilization | *hemO* | + | + | + | + |
|  | Achromobactin biosynthesis and transport | *cbrD* | + | - | - | - |
|  | Heme biosynthesis | *hemL* | + | - | - | - |
|  | Periplasmic binding protein-dependent ABC transport systems | *vctC* | + | - | - | - |
| Regulation | Quorum sensing | *abaI* | + | + | + | + |
|  |  | *abaR* | + | + | + | + |
|  | Two-component system | *bfmR* | + | + | + | + |
|  |  | *bfmS* | + | + | + | + |
| Cell surface components | Trehalose-recycling ABC transporter | *sugC* | + | - | - | - |
| Copper uptake | Copper exporter | *ctpV* | + | - | - | - |
| Intracellular survival | Lipoate protein ligase A1 | *lplA1* | + | - | - | - |
| Invasion | Lipoprotein promoting entry protein | *lpeA* | + | - | - | - |
| Lipid and fatty acid metabolism | Panthothenate synthesis | *panD* | + | - | - | - |
| Peptidoglycan modification | OatA | *oatA* | + | - | - | - |
| Secretion system | Type III secretion system | *cdsN* | + | - | - | - |
| Toxin | Hemolysin III | *hlyIII* | + | - | - | - |
| Serum resistance | PbpG | *pbpG* | + | + | + | + |
| Stress adaption | Catalase | *katA* | + | + | + | + |

**Supplementary Table 5.** Biofilm formation of *Acinetobacter baumannii* clinical isolates

| **Strain number** | **Origin of the strain** | **Cut-off value (ODc)** | **OD average** | **OD (OD average - ODc)** | **Biofilm formation category** |
| --- | --- | --- | --- | --- | --- |
| 1 | hemoculture | 0.2881 | 0.8250 | 0.5370 | weak |
| 2 | tip of the aspirator | 0.2881 | 1.6957 | 1.4077 | strong |
| 3 | tip of the aspirator | 0.2881 | 2.4116 | 2.1235 | strong |
| 4 | tip of the aspirator | 0.2881 | 1.9408 | 1.6527 | strong |
| 5 | hemoculture | 0.2881 | 1.9806 | 1.6925 | strong |
| 6 | hemoculture | 0.2881 | 1.9579 | 1.6698 | strong |
| 7 | hemoculture | 0.2881 | 2.3482 | 2.0602 | strong |
| 8 | tracheal aspirate | 0.2881 | 2.1134 | 1.8254 | strong |
| 9 | tracheal aspirate | 0.2881 | 1.7030 | 1.4150 | strong |
| 10 | tracheal aspirate | 0.2881 | 2.3746 | 2.0866 | strong |
| 11 | tracheal aspirate | 0.2881 | 2.3485 | 2.0604 | strong |
| 12 | hemoculture | 0.2881 | 1.9658 | 1.6778 | strong |
| 13 | hemoculture | 0.2881 | 1.9613 | 1.6732 | strong |
| 14 | hemoculture | 0.2881 | 2.0312 | 1.7431 | strong |
| 15 | hemoculture | 0.2881 | 1.8224 | 1.5343 | strong |
| 16 | hemoculture | 0.2881 | 2.1890 | 1.9009 | strong |
| 17 | hemoculture | 0.2881 | 1.7697 | 1.4816 | strong |
| 18 | hemoculture | 0.2881 | 2.1643 | 1.8762 | strong |
| 19 | hemoculture | 0.2881 | 1.8153 | 1.5272 | strong |
| 20 | hemoculture | 0.2881 | 2.7972 | 2.5091 | strong |
| 21 | tracheal aspirate | 0.2881 | 2.3646 | 2.0765 | strong |
| 22 | tip of the central venous aspirator | 0.2733 | 0.8720 | 0.5987 | moderate |
| 23 | tip of the central venous aspirator | 0.2733 | 0.9776 | 0.7042 | moderate |
| 24 | tip of the central venous aspirator | 0.2733 | 1.0587 | 0.7854 | moderate |
| 25 | tip of the central venous aspirator | 0.3028 | 3.7244 | 3.4217 | strong |
| 26 | tip of the central venous aspirator | 0.3028 | 3.6975 | 3.3947 | strong |
| 27 | hemoculture | 0.3028 | 0.9880 | 0.6853 | moderate |
| 28 | hemoculture | 0.3028 | 0.9224 | 0.6196 | moderate |
| 29 | hemoculture | 0.3028 | 1.1070 | 0.8042 | moderate |
| 30 | hemoculture | 0.3028 | 1.5864 | 1.2837 | strong |
| 31 | tip of the central venous aspirator | 0.3028 | 1.7434 | 1.4406 | strong |
| 32 | tip of the central venous aspirator | 0.3028 | 1.1289 | 0.8261 | moderate |
| 33 | tracheal aspirate | 0.3028 | 1.5576 | 1.2548 | strong |
| 34 | tip of the central venous aspirator | 0.3028 | 1.6950 | 1.3922 | strong |
| 35 | tip of the central venous aspirator | 0.3028 | 1.2632 | 0.9605 | moderate |
| 36 | tracheal aspirate | 0.3028 | 1.5505 | 1.2478 | strong |
| 37 | sputum | 0.3028 | 2.3367 | 2.0340 | strong |
| 38 | tip of the central venous aspirator | 0.3028 | 2.4170 | 2.1142 | strong |
| 39 | tip of the central venous aspirator | 0.3028 | 2.1251 | 1.8223 | strong |
| 40 | hemoculture | 0.3028 | 1.8384 | 1.5356 | strong |
| 41 | hemoculture | 0.3028 | 1.9977 | 1.6949 | strong |
| 42 | hemoculture | 0.3028 | 1.3416 | 1.0388 | moderate |
| 43 | hemoculture | 0.3028 | 1.5961 | 1.2933 | strong |
| 44 | hemoculture | 0.3028 | 1.6231 | 1.3204 | strong |
| 45 | hemoculture | 0.3028 | 1.5799 | 1.2771 | strong |
| 46 | tip of the central venous aspirator | 0.3028 | 1.5455 | 1.2427 | strong |
| 47 | tip of the central venous aspirator | 0.3028 | 1.4531 | 1.1504 | moderate |
| 48 | tip of the central venous aspirator | 0.3028 | 1.1765 | 0.8737 | moderate |
| 49 | hemoculture | 0.3028 | 2.3577 | 2.0549 | strong |
| 50 | tip of the central venous aspirator | 0.3028 | 1.7024 | 1.3997 | strong |
| 51 | tip of the central venous aspirator | 0.3028 | 1.7245 | 1.4218 | strong |
| 52 | tip of the central venous aspirator | 0.3028 | 1.5301 | 1.2274 | strong |
| 53 | tip of the central venous aspirator | 0.2683 | 0.8066 | 0.5383 | moderate |
| 54 | hemoculture | 0.2683 | 1.6114 | 1.3431 | strong |
| 55 | hemoculture | 0.2683 | 1.1942 | 0.9258 | moderate |
| 56 | hemoculture | 0.2683 | 1.3546 | 1.0862 | strong |
| 57 | tracheal aspirate | 0.2683 | 1.6059 | 1.3376 | strong |
| 58 | tip of the central venous aspirator | 0.2683 | 1.4415 | 1.1732 | strong |
| 59 | tip of the central venous aspirator | 0.2683 | 1.4399 | 1.1716 | strong |
| 60 | hemoculture | 0.2683 | 1.2504 | 0.9820 | moderate |
| 61 | tracheal aspirate | 0.2683 | 1.7658 | 1.4974 | strong |
| 62 | tracheal aspirate | 0.2683 | 1.5551 | 1.2867 | strong |
| 63 | tip of the central venous aspirator | 0.2683 | 1.1536 | 0.8853 | moderate |
| 64 | tip of the central venous aspirator | 0.2683 | 1.5611 | 1.2928 | strong |

**Supplementary Table 6.** Duration of patient hospitalization at ICU and days of hospitalization at ICU prior to *A. baumannii* isolation.

| Isolate | Date of patient hospitalization at ICU | Date of the patient's discharge | Duration of hospitalization at ICU (days) | Sample collection | Duration of hospitalization prior *A. baumannii* isolation (days) | Type of the sample |
| --- | --- | --- | --- | --- | --- | --- |
| 1 | 2020-12-23 | 2021-01-13 | 21 | 2021-01-01 | 9 | hemoculture |
| 2 | 2020-12-29 | 2021-01-09 | 11 | 2021-01-01 | 3 | tip of the aspirator |
| 3 | 2020-12-23 | 2021-01-13 | 21 | 2021-01-01 | 9 | tip of the aspirator |
| 4 | 2020-12-10 | 2021-01-02 | 23 | 2021-01-01 | 22 | tip of the aspirator |
| 5 | 2020-12-08 | 2020-12-31 | 23 | 2020-12-31 | 24 | hemoculture |
| 6 | 2020-12-30 | 2021-01-04 | 5 | 2020-12-31 | 1 | hemoculture |
| 7 | 2020-12-26 | 2021-01-10 | 15 | 2021-12-31 | 5 | hemoculture |
| 8 | 2020-12-08 | 2020-12-31 | 23 | 2020-12-31 | 23 | TBA* |
| 9 | 2020-12-25 | 2021-01-03 | 9 | 2020-12-31 | 6 | TBA* |
| 10 | 2020-12-28 | 2021-01-03 | 6 | 2021-12-31 | 3 | TBA* |
| 11 | 2020-12-23 | 2021-01-13 | 21 | 2020-12-31 | 8 | TBA* |
| 12 | 2020-12-29 | 2021-01-06 | 8 | 2021-01-04 | 6 | hemoculture |
| 13 | 2020-12-29 | 2021-01-06 | 8 | 2021-01-05 | 7 | hemoculture |
| 14 | 2020-12-31 | 2021-01-12 | 12 | 2021-01-07 | 7 | hemoculture |
| 15 | 2020-12-23 | 2021-01-13 | 21 | 2021-01-07 | 15 | hemoculture |
| 16 | 2020-12-26 | 2021-01-10 | 15 | 2021-01-08 | 13 | hemoculture |
| 17 | 2020-12-26 | 2021-01-10 | 15 | 2021-01-08 | 13 | hemoculture |
| 18 | 2021-01-04 | 2021-01-20 | 16 | 2021-01-11 | 7 | hemoculture |
| 19 | 2021-01-04 | 2021-01-20 | 16 | 2021-01-11 | 7 | hemoculture |
| 20 | 2020-12-31 | 2021-01-12 | 12 | 2021-01-11 | 11 | hemoculture |
| 21 | 2020-12-29 | 2021-01-06 | 8 | 2021-01-04 | 6 | TBA* |
| 22 | 2020-12-31 | 2021-02-10 | 41 | 2021-01-07 | 7 | CVC** |
| 23 | 2020-12-31 | 2021-01-12 | 12 | 2021-01-08 | 8 | CVC** |
| 24 | 2020-12-23 | 2021-01-13 | 21 | 2021-01-07 | 15 | CVC** |
| 25 | 2020-12-23 | 2021-01-13 | 21 | 2021-01-09 | 17 | CVC** |
| 26 | 2020-12-31 | 2021-01-12 | 12 | 2021-01-11 | 11 | CVC** |
| 27 | 2021-01-10 | 2021-01-22 | 12 | 2021-01-14 | 4 | hemoculture |
| 28 | 2021-01-10 | 2021-01-22 | 12 | 2021-01-15 | 5 | hemoculture |
| 29 | 2021-01-10 | 2021-01-22 | 12 | 2021-01-15 | 5 | hemoculture |
| 30 | 2021-01-08 | 2021-01-18 | 10 | 2021-01-16 | 8 | hemoculture |
| 31 | 2021-01-04 | 2021-01-20 | 16 | 2021-01-11 | 7 | CVC** |
| 32 | 2021-01-10 | 2021-01-22 | 12 | 2021-01-14 | 4 | CVC** |
| 33 | 2021-01-08 | 2021-01-18 | 10 | 2021-01-15 | 7 | TBA* |
| 34 | 2021-01-10 | 2021-01-22 | 12 | 2021-01-16 | 6 | CVC** |
| 35 | 2021-01-08 | 2021-01-26 | 18 | 2021-01-16 | 8 | CVC** |
| 36 | 2021-01-24 | 2021-03-11 | 46 | 2021-01-27 | 3 | TBA* |
| 37 | 2021-01-24 | 2021-03-11 | 46 | 2021-01-29 | 5 | sputum |
| 38 | 2021-01-16 | 2021-01-28 | 12 | 2021-01-26 | 10 | CVC** |
| 39 | 2021-01-25 | 2021-02-02 | 8 | 2021-01-31 | 6 | CVC** |
| 40 | 2021-01-12 | 2021-01-20 | 8 | 2021-01-18 | 6 | hemoculture |
| 41 | 2021-01-12 | 2021-01-20 | 8 | 2021-01-18 | 6 | hemoculture |
| 42 | 2021-01-04 | 2021-01-20 | 16 | 2021-01-19 | 15 | hemoculture |
| 43 | 2020-12-31 | 2021-01-26 | 26 | 2021-01-22 | 22 | hemoculture |
| 44 | 2021-01-12 | 2021-02-04 | 23 | 2021-01-23 | 11 | hemoculture |
| 45 | 2021-01-12 | 2021-02-04 | 23 | 2021-01-23 | 11 | hemoculture |
| 46 | 2021-01-12 | 2021-02-04 | 23 | 2021-01-19 | 7 | CVC** |
| 47 | 2021-01-04 | 2021-01-20 | 16 | 2021-01-19 | 15 | CVC** |
| 48 | 2021-01-11 | 2021-01-20 | 9 | 2021-01-19 | 8 | CVC** |
| 49 | 2021-01-12 | 2021-02-04 | 23 | 2021-01-25 | 13 | hemoculture |
| 50 | 2021-01-02 | 2021-01-24 | 22 | 2021-01-22 | 20 | CVC** |
| 51 | 2021-01-08 | 2021-01-30 | 22 | 2021-01-23 | 15 | CVC** |
| 52 | 2021-01-12 | 2021-02-04 | 23 | 2021-01-25 | 13 | CVC** |
| 53 | 2021-01-15 | 2021-01-27 | 12 | 2021-01-25 | 10 | CVC** |
| 54 | 2021-01-10 | 2021-01-30 | 20 | 2021-01-30 | 20 | hemoculture |
| 55 | 2021-01-10 | 2021-01-30 | 20 | 2021-01-29 | 19 | hemoculture |
| 56 | 2021-01-25 | 2021-02-02 | 8 | 2021-01-30 | 5 | hemoculture |
| 57 | 2021-01-18 | 2021-02-01 | 14 | 2021-01-27 | 9 | TBA* |
| 58 | 2021-01-07 | 2021-01-29 | 22 | 2021-01-28 | 21 | CVC** |
| 59 | 2021-01-18 | 2021-02-01 | 14 | 2021-01-31 | 13 | CVC** |
| 60 | 2021-01-12 | 2021-02-04 | 23 | 2021-02-03 | 22 | TBA* |
| 61 | 2021-01-12 | 2021-02-04 | 23 | 2021-02-03 | 22 | TBA* |
| 62 | 2021-01-30 | 2021-02-18 | 19 | 2021-02-03 | 4 | TBA* |
| 63 | 2021-02-01 | 2021-02-11 | 10 | 2021-02-08 | 7 | CVC** |
| 64 | 2021-02-01 | 2021-02-11 | 10 | 2021-02-09 | 8 | CVC** |

TBA^*^- tracheal aspirate; CVC^**^- tip of the central venous catheter

# Supplementary Figures

**Supplementary Figure 1.** Twitching motility distribution of *A. baumannii* isolates.


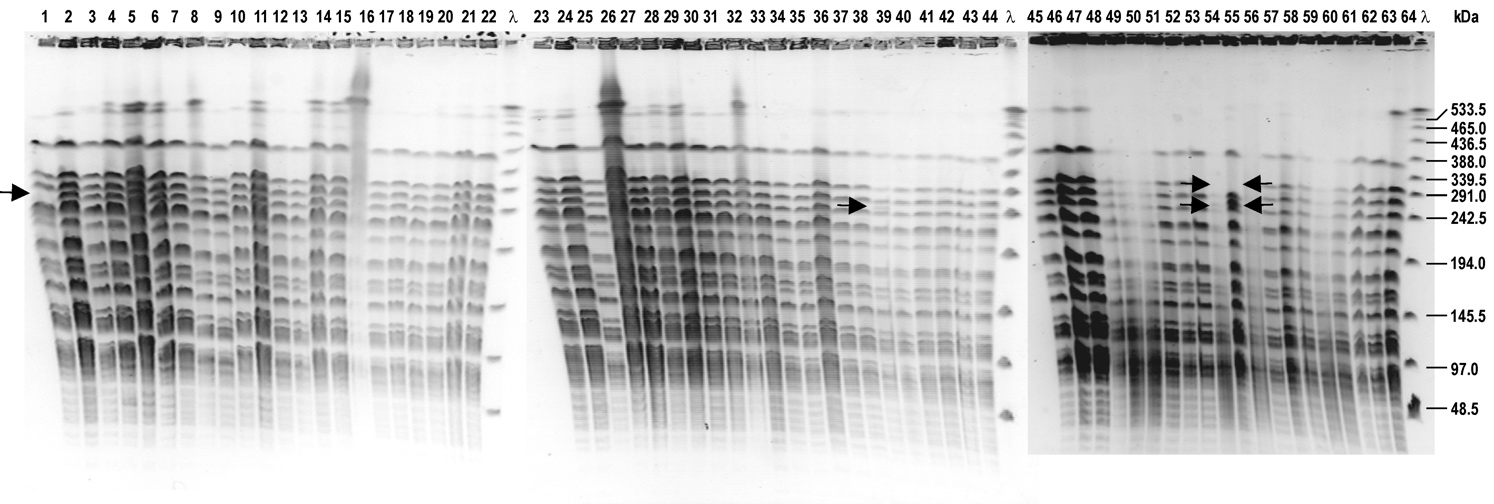


**Supplementary Figure 2.** PFGE profiles of *A. baumannii* isolates from COVID-19 patients admitted to ICU at General Hospital “Dr Laza K. Lazarević” Šabac, Serbia (1-64), obtained by *Apa*I digestion. Arrows indicate that isolate 1 lacks an *Apa*I fragment of 242 kb; isolate 39 has an extra *Apa*I fragment of 250 kb; isolates 54 and 55 have the same profile, differing from others in that they lack a fragment of 300 kb, and have a new unique fragment of 250 kb. λ - λ concatemers (New England Biolabs)

**a.**


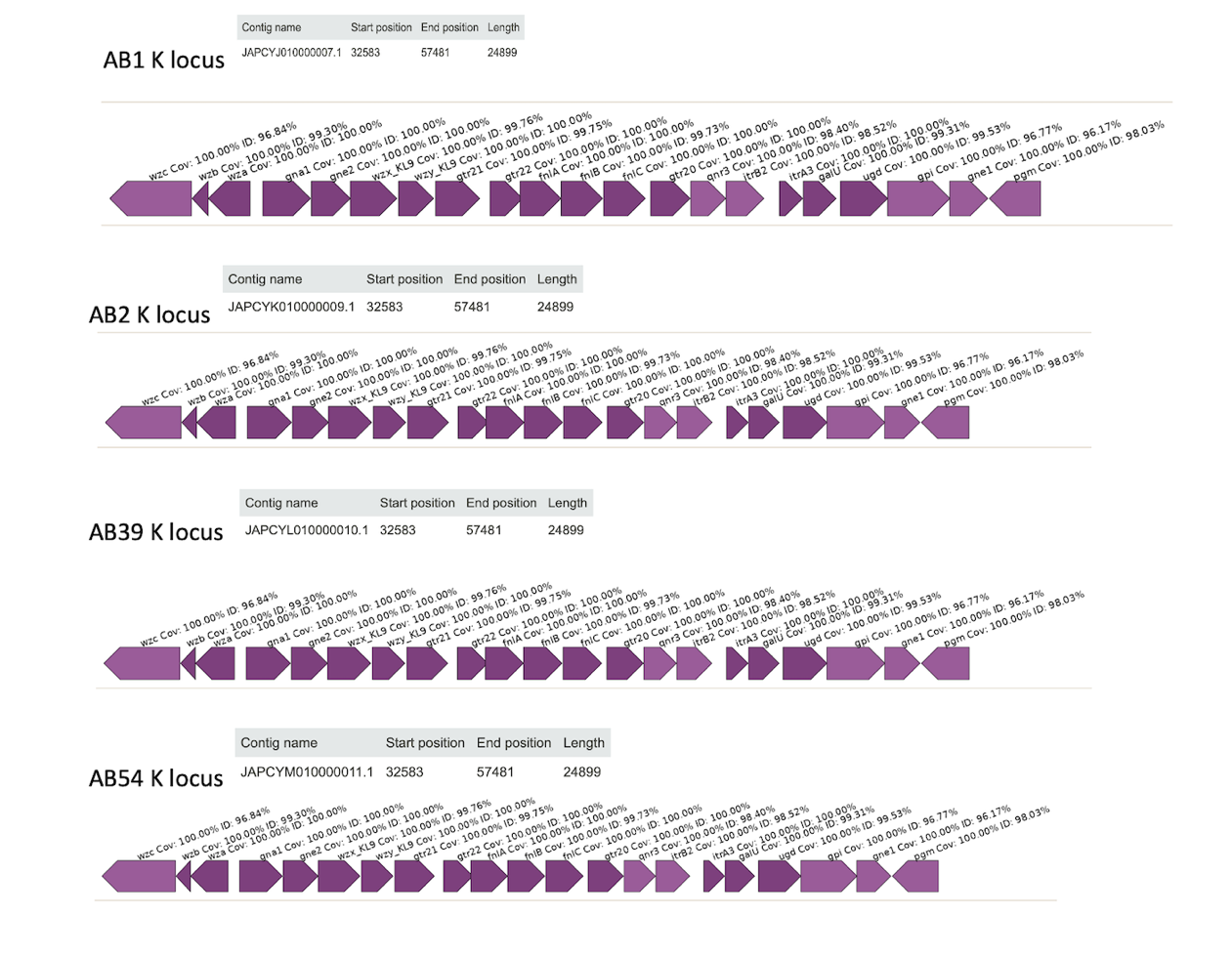


**b.**

**
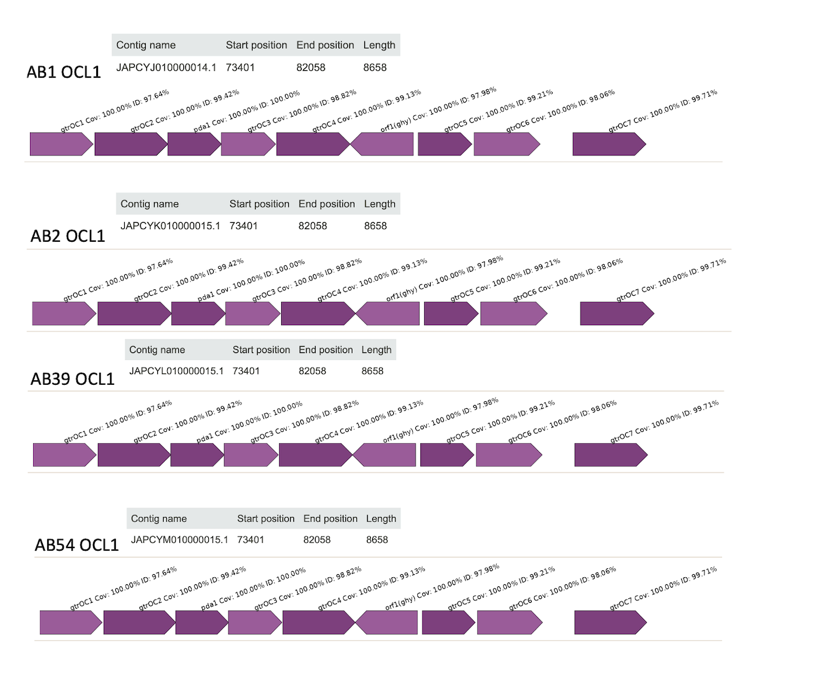
**

**Supplementary Figure 3.** Comparisson of K locus (3a) and outer LPS (O) locus (3b) using Kaptive 2.0 tool.


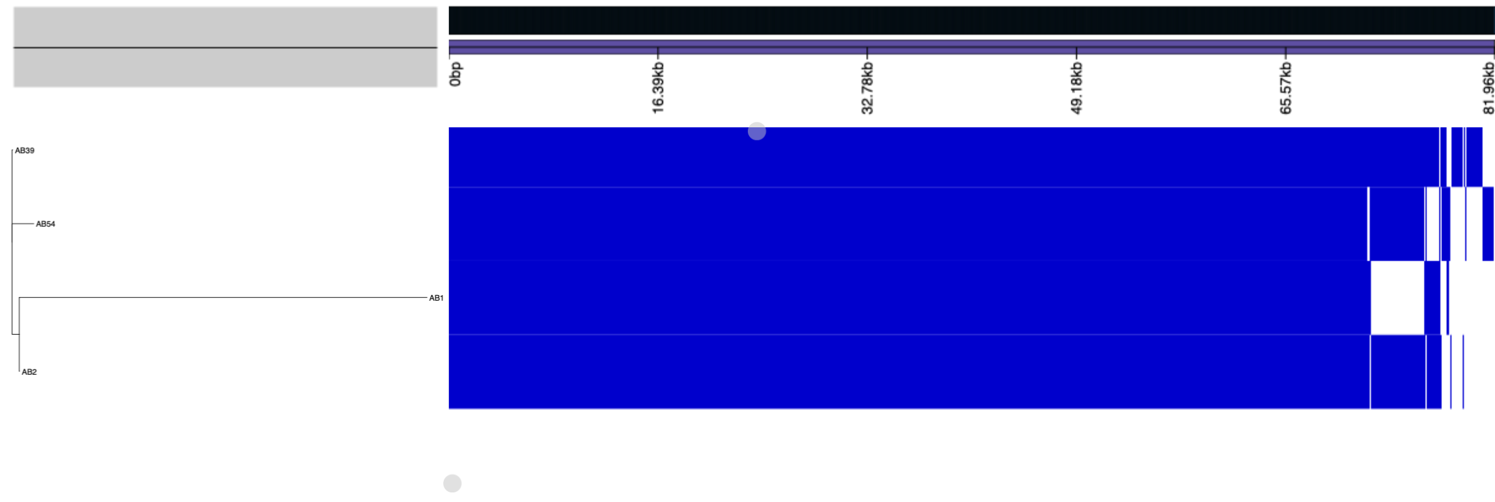


**Supplementary Figure 4.** Visualization of pan-genome constructed using Roary based on the core and accessory genes showing phylogenetic relatedness of the isolates by blue (present) and white (absent) fragments.


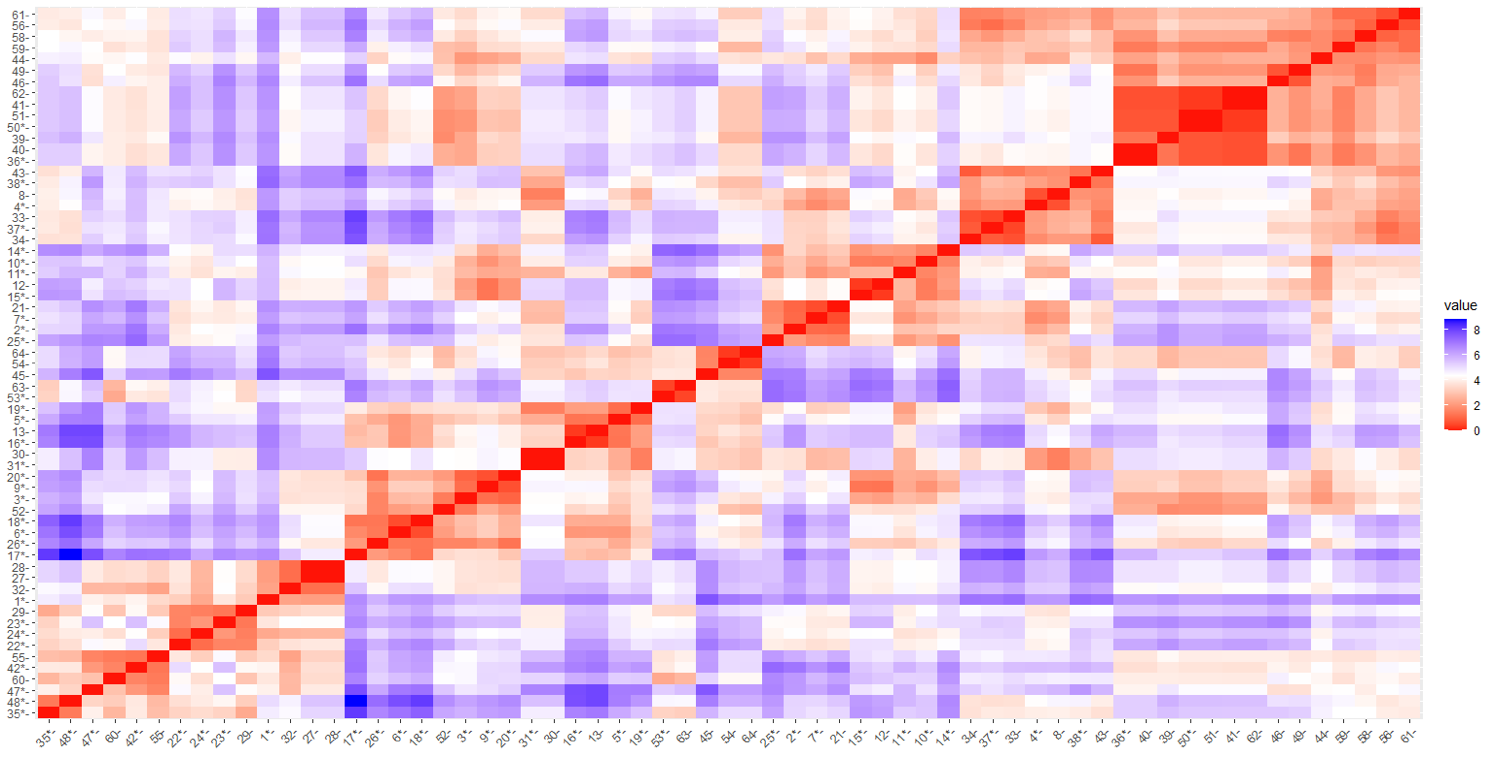


**Supplementary Figure 5.** Heatmap displaying the results of hierarchical clustering among 64 *A.baumannii* isolates. The color scale indicates the degree of correlation (blue, low correlation; red, high correlation).
